# Supplementary material for: Urban–rural disparity in cancer incidence in China, 2008–2012: a cross-sectional analysis of data from 36 cancer registers
Source: BMJ Open. 2021 Apr 30;11(4):e042762. doi: 10.1136/bmjopen-2020-042762 (PMC8098914; doi:10.1136/bmjopen-2020-042762)
Supplement: Supplementary data [file bmjopen-2020-042762supp001.pdf]

BMJ Publishing Group Limited (BMJ) disclaims all liability and responsibility arising from any reliance placed on this supplemental material which has been supplied by the author(s)

Supporting information for

Urban-rural disparity in cancer incidence in China, 2008-2012: A cross-sectional analysis of data from 36 cancer registers

Shuai Yuan, Shao-Hua Xie

Supplementary Table 1. Chinese cancer registries in the Cancer Incidence in Five Continents, XI volume

Supplementary Table 2. Data quality of Chinese cancer registries in the Cancer Incidence in Five Continents, XI volume

Supplementary Table 1. Chinese cancer registries in the Cancer Incidence in Five Continents, XI volume

Region	Registry	Calendar period
Rural areas		
North	Cixian County	2008-2012
North	Shexian County	2008-2012
East	Qidong County	2008-2012
East	Jiashan County	2008-2012
East	Haimen County	2008-2012
East	Jianhu County	2010-2012
East	Guanyun	2008-2012
East	Sheyang	2008-2012
East	Xianju	2010-2012
Central	Linzhou County	2008-2012
Central	Hengdong	2009-2012
Central	Xiping	2010-2012
Central	Yanshi	2010-2012
Southwest	Yanting County	2008-2012
Urban areas		
North	Beijing	2008-2012
Northeast	Anshan City	2008-2012
Northeast	Harbin City, Nangang District	2008-2012
Northeast	Benxi	2008-2011
Northeast	Shenyang	2008-2012
East	Shanghai City	2008-2012
East	Hangzhou City	2008-2012
East	Jiaxing City	2008-2012
East	Hefei	2010-2012
East	Huaiyin District, Huai'an	2009-2012
East	Lianyungang	2008-2012
East	Maanshan	2008-2012
East	Tongling City	2008-2012
East	Wuxi	2010-2012
Central	Wuhan City	2008-2012
Central	Yueyanglou	2009-2012
South	Zhongshan City	2010-2012
South	Guangzhou	2010-2012
South	Hong Kong	2008-2012
South	Jiangmen	2010-2012
South	Liuzhou	2009-2012
South	Zhuhai	2010-2012

Supplementary Table 2. Data quality of Chinese cancer registries in the Cancer Incidence in Five Continents, XI volume

Region		Male				Female			
		Cases	MV (%)	DCO (%)	ASR (W)	Cases	MV (%)	DCO (%)	ASR (W)
**Rural areas**									
North	Cixian County	5,160	79.7	2.5	443.2	3,765	78.5	1.7	280.7
North	Shexian County	3,839	74.0	1.9	343.5	2,297	77.2	2.3	201.9
East	Qidong County	11,544	44.2	0.0	218.4	7,661	58.2	0.0	132.4
East	Jiashan County	4,068	64.5	0.1	224.1	2,999	73.2	0.0	169.4
East	Haimen County	10,521	48.4	0.5	209.6	7,623	62.8	0.5	154.6
East	Jianhu County	4,241	76.9	0.1	253.0	2,792	82.5	0.3	162.4
East	Guanyun	5,924	51.7	1.8	181.6	4,119	65.6	2.1	137.3
East	Sheyang	8,647	63.2	0.3	225.0	6,415	70.7	0.3	168.0
East	Xianju	2,983	64.8	4.5	277.4	1,780	69.8	4.2	174.7
Central	Linzhou County	8,721	78.4	1.5	309.8	7,111	78.2	1.3	220.5
Central	Hengdong	3,266	60.4	7.2	171.8	2,382	71.2	5.0	131.7
Central	Xiping	3,050	63.3	2.5	200.5	2,550	70.3	1.8	160.9
Central	Yanshi	2,167	67.9	3.9	215.2	1,969	69.4	4.2	170.7
Southwest	Yanting County	7,053	77.9	1.4	435.6	4,560	79.5	2.1	256.6
**Urban areas**									
North	Beijing	61,058	71.4	1.9	166.7	58,686	79.1	1.4	165.6
Northeast	Anshan City	12,562	62.4	11.8	210.5	11,581	72.6	10.5	178.8
Northeast	Harbin City, Nangang District	6,509	70.5	3.9	191.9	6,009	78.7	2.5	154.9
Northeast	Benxi	5,171	52.2	6.1	244.0	4,294	64.3	4.3	181.4
Northeast	Shenyang	26,978	61.1	6.8	181.5	25,643	69.9	5.0	157.4
East	Shanghai City	68,195	69.8	0.0	200.9	62,499	75.9	0.0	187.6
East	Hangzhou City	55,665	66.1	0.7	206.2	46,116	73.4	0.5	176.3
East	Jiaxing City	4,605	64.7	0.1	207.1	3,923	75.6	0.2	177.8
East	Hefei	10,092	52.5	8.1	252.7	6,268	58.8	6.2	155.7
East	Huaiyin District, Huai'an	5,846	62.2	8.3	220.7	3,406	70.0	7.8	130.3
East	Lianyungang	5,776	67.9	1.5	185.9	4,422	74.9	1.2	138.2
East	Maanshan	5,417	69.0	0.4	268.8	3,567	76.4	0.5	173.1
East	Tongling City	4,076	75.3	5.0	322.5	2,315	78.0	4.0	174.9
East	Wuxi	13,086	63.0	1.9	205.1	9,544	71.0	1.6	149.2
Central	Wuhan City	36,737	71.0	0.7	205.1	30,701	79.7	0.6	167.6
Central	Yueyanglou	2,396	76.1	2.9	281.0	1,795	82.5	2.8	190.8
South	Zhongshan City	6,828	70.9	0.0	251.9	4,776	83.0	0.0	158.4
South	Guangzhou	33,619	70.0	0.8	204.1	27,801	82.0	0.4	162.2
South	Hong Kong	66,837	84.0	0.7	229.8	60,084	89.7	0.6	189.9
South	Jiangmen	2,890	69.1	0.6	236.3	2,249	83.0	0.5	170.1
South	Liuzhou	5,825	62.3	2.7	224.2	4,437	75.2	2.1	165.5
South	Zhuhai	4,155	62.3	3.7	243.8	3,450	72.2	2.4	194.1

ASR, age-standardized rate; DCO, death certificate only; MV, microscopically verified.
