# Supplementary material for: Urban–rural disparity in cancer incidence in China, 2008–2012: a cross-sectional analysis of data from 36 cancer registers
Source: BMJ Open. 2021 Apr 30;11(4):e042762. doi: 10.1136/bmjopen-2020-042762 (PMC8098914; doi:10.1136/bmjopen-2020-042762)
Supplement: Author's manuscript [file bmjopen-2020-042762.draft_revisions.pdf]

BMJ Open

BMJ Open is committed to open peer review. As part of this commitment we make the peer review history of every article we publish publicly available.

When an article is published we post the peer reviewers' comments and the authors' responses online. We also post the versions of the paper that were used during peer review. These are the versions that the peer review comments apply to.

The versions of the paper that follow are the versions that were submitted during the peer review process. They are not the versions of record or the final published versions. They should not be cited or distributed as the published version of this manuscript.

BMJ Open is an open access journal and the full, final, typeset and author-corrected version of record of the manuscript is available on our site with no access controls, subscription charges or pay-per-view fees (http://bmjopen.bmj.com).

If you have any questions on BMJ Open's open peer review process please email info.bmjopen@bmj.com

BMJ Open

Urban-rural disparity in cancer incidence in China, 2008-2012

Journal:	*BMJ Open*
Manuscript ID	bmjopen-2020-042762
Article Type:	Original research
Date Submitted by the Author:	15-Jul-2020
Complete List of Authors:	Yuan, Shuai; Karolinska Institutet, Institute of Environmental Medicine<br>Xie, Shao-Hua; Karolinska Institutet, Department of Molecular Medicine and Surgery
Keywords:	EPIDEMIOLOGY, ONCOLOGY, PUBLIC HEALTH, SOCIAL MEDICINE

SCHOLARONE™
Manuscripts

BMJ

I, the Submitting Author has the right to grant and does grant on behalf of all authors of the Work (as defined in the below author licence), an exclusive licence and/or a non-exclusive licence for contributions from authors who are: i) UK Crown employees; ii) where BMJ has agreed a CC-BY licence shall apply, and/or iii) in accordance with the terms applicable for US Federal Government officers or employees acting as part of their official duties; on a worldwide, perpetual, irrevocable, royalty-free basis to BMJ Publishing Group Ltd ("BMJ") its licensees and where the relevant Journal is co-owned by BMJ to the co-owners of the Journal, to publish the Work in this journal and any other BMJ products and to exploit all rights, as set out in our licence.

The Submitting Author accepts and understands that any supply made under these terms is made by BMJ to the Submitting Author unless you are acting as an employee on behalf of your employer or a postgraduate student of an affiliated institution which is paying any applicable article publishing charge ("APC") for Open Access articles. Where the Submitting Author wishes to make the Work available on an Open Access basis (and intends to pay the relevant APC), the terms of reuse of such Open Access shall be governed by a Creative Commons licence – details of these licences and which Creative Commons licence will apply to this Work are set out in our licence referred to above.

Other than as permitted in any relevant BMJ Author's Self Archiving Policies, I confirm this Work has not been accepted for publication elsewhere, is not being considered for publication elsewhere and does not duplicate material already published. I confirm all authors consent to publication of this Work and authorise the granting of this licence.

For peer review only

Title: Urban-rural disparity in cancer incidence in China, 2008-2012

Running head: Cancer disparity in China

Authors: Shuai Yuan, MD, MSc[1], Shao-Hua Xie, MD, PhD[2*]

Affiliations

[1] Unit of Cardiovascular and Nutritional Epidemiology, Institute of Environmental Medicine, Karolinska Institutet, Stockholm, Sweden. shuai.yuan@ki.se

[2] Upper Gastrointestinal Surgery, Department of Molecular Medicine and Surgery, Karolinska Institutet, Karolinska University Hospital, Stockholm, Sweden shaohua.xie@ki.se

[*]**Correspondence**

Shao-Hua Xie, MD, PhD, Upper Gastrointestinal Surgery, Department of Molecular medicine and Surgery, Karolinska Institutet, Karolinska University Hospital, Retzius väg 13a, 4th Floor, 17 177 Stockholm, Sweden. Email: shaohua.xie@ki.se.

Words of Abstract / Manuscript: 226 / 2390

Number of tables / figures: 3 / 2

For peer review only - http://bmjopen.bmj.com/site/about/guidelines.xhtml

Strengths and limitations of this study

- One major strength was that we used data from the 36 cancer registers of high quality in terms of completeness of coverage and accuracy, thereby lending validity to the findings.
- We comprehensively investigated urban-rural disparity for overall and site-specific cancer incidence by sex and on both absolute and relative scales.
- One limitation is that the included registries only represented a limited proportion of the total population in China and were not in well balanced distribution regarding geographic regions, and thus, the strengths of the disparity measures may not exactly reflect the extent of nationwide urban-rural disparity in cancer incidence.
- Due to unavailability of complete data in all cancer registries, we were not able to analyse the data by histological type.
- The analyses were based on cancer incidence data only without information on risk factors, and we could not direct evaluate how the observed disparity could be explained by the corresponding risk factors.

For peer review only

Abstract

Objective: The substantial differences in socioeconomic and lifestyle exposures between urban and rural areas in China may lead to urban-rural disparity in cancer risk. This study aimed to assess the urban-rural disparity in cancer incidence in China.

Methods: Using data from 36 regional cancer registries in China in 2008-2012, we compared the age-standardized incidence rates of cancer by sex and anatomic site between rural and urban areas. We calculated the rate difference (RD) and rate ratio (RR) comparing rates in rural versus urban areas by sex and cancer type.

Results The incidence rate of all cancers in women was slightly lower in rural areas than in rural areas (RD=-4.7 per 100,000 person-years), but the total cancer rate in men was higher in rural areas than in urban areas (RD=39.9 per 100,000 person-years). The incidence rates in women were higher in rural areas than in urban areas for cancers of the esophagus (RD=23.6 per 100,000 person-years; RR=9.8), stomach (RD=16.0 per 100,000 person-years; RR=2.5), and liver and biliary passages (RD=8.0 per 100,000 person-years; RR=1.7), but lower for cancers of thyroid (RD=-9.6 per 100,000 person-years; RR=0.3) and breast (RD=-19.8 per 100,000 person-years; RR=0.5). Men residing in rural areas had higher incidence rates for cancers of the esophagus (RD=32.3 per 100,000 person-years; RR=3.9), stomach (RD=38.4 per 100,000 person-years; RR=2.7), and liver and biliary passages (RD=16.8 per 100,000 person-years; RR=1.6), but lower rates for prostate cancer (RD=-9.0 per 100,000 person-years; RR=0.3), lip, oral cavity and pharynx cancer (RD=-5.1 per 100,000 person-years; RR=0.5), and colorectal cancer (RD=-13.6 per 100,000 person-years; RR=0.6) .

For peer review only - http://bmjopen.bmj.com/site/about/guidelines.xhtml

Conclusions: Our findings suggest substantial urban-rural disparity in cancer incidence in China, which varies across cancer types and the sexes. Cancer prevention strategies should be tailored for common cancers in rural and urban areas.

Keywords: Neoplasms; incidence; socioeconomic status; spatial distribution; epidemiology

For peer review only

For peer review only - http://bmjopen.bmj.com/site/about/guidelines.xhtml

1 INTRODUCTION

Cancer is one of the leading causes of death worldwide, including in China. According to China Health Statistics Yearbook, an estimated 2.2 million cancer deaths occurred in China in 2017, accounting for around 25% of all deaths in this year [1]. Moreover, the past few decades have witnessed rapid urbanization and industrialization in China, and the uneven distribution of wealth and lifestyle profiles across regions in the nation during this process may have in turn resulted in substantial urban-rural disparity in cancer risk. Previous studies have suggested marked urban-rural disparity in cancer incidence in China, and such disparity seems varying across cancer types [2-8]. However, most of these previous studies were limited in a certain area or province, or specific types of cancer only. Due to historically limited population-based cancer registry data in China, the urban-rural disparity in cancer incidence has not been well characterized.

China has recently established the National Central Cancer Registry and data from 36 regional cancer registries have been included in the latest release of *Cancer Incidence in Five Continents* series published by the International Agency for Research on Cancer [9]. This provides a new opportunity to assess the regional disparity in cancer incidence in China on a national scale. Therefore, using data from these high-quality registries, we conducted the present study to characterize the urban-rural disparity in total and type-specific cancer incidence by sex in China in 2008-2012.

2 MATERIALS AND METHODS

2.1 Data source

For peer review only - http://bmjopen.bmj.com/site/about/guidelines.xhtml

We extracted data on cancer incidence and population sizes from 36 regional cancer registries, including 14 registries in rural areas and 22 in urban areas, in China during 2008-2012 which are included in the XI volume of *Cancer Incidence in Five Continents* series published by the International Agency for Research on Cancer [10]. These registries are located in different geographical regions throughout the country. Detailed information on the included registries is presented in **Table 1**. We pooled the numbers of cases and population sizes at risk from multiple registers separately for urban and rural areas by sex and cancer type (anatomic site).

2.2 Statistical analysis

We calculated the crude and age-standardized incidence rates (ASRs) and their 95% confidence intervals (CIs) by sex and cancer type separately for rural and urban areas. The ASRs were computed by using the direct standardization method with World Standard Population 2000 as reference [11]. The 95% CIs of crude rates were estimated under the assumption of Poisson distribution and CIs for ASRs were calculated based on the gamma distribution assuming that the standardized rate is a weighted sum of independent Poisson random variables [12].

The urban-rural disparities in cancer incidence were quantitatively assessed with two disparity measures, i.e. rate difference (RD) on the absolute scale and rate ratio (RR) on the relative scale [13]. We calculated the RD (ASR in rural areas – ASR in urban areas) and rate ratio (ratio of ASRs in rural areas relative to urban areas) with 95% CIs by sex and cancer type. All statistical analyses were performed using the SAS version 9.4 (SAS Institute, Cary, NC) and two-sided.

2.3 Patient and Public Involvement

No patient involved.

For peer review only - http://bmjopen.bmj.com/site/about/guidelines.xhtml

3 RESULTS

3.1 Cancer incidence in rural areas

The crude rates and ASRs in rural and urban areas are displayed in **Table 2** for women **and in Table 3** for men. In total, 58 576 female and 81 709 male new cancer cases were recorded in 14 cancer registers in rural areas in China during the period 2008-2012, contributing to the ASRs of 188.6 (95% CI 187.1-190.2) per 100 000 person-years in women and 273.4 (95% CI 271.5-275.2) per 100 000 person-years in men. In women residing in rural areas, the highest ASRs were observed for stomach cancer (26.5 per 100 000 person-years; 95% CI 25.9-27.1), followed by cancers of the esophagus (26.3; 95% CI, 25.8-26.9), lung (22.6; 95% CI, 22.0-23.1), breast (21.5; 95% CI, 21.0-22.0), and liver and biliary passages (19.2; 95% CI, 18.8-19.7). In men in rural areas, stomach cancer was also the most common cancer type (ASR 61.3; 95% CI, 60.4-62.2) , followed by cancers of the lung (52.7; 95% CI, 51.9-53.5), liver and biliary passages (45.8; 95% CI, 45.1-46.6), esophagus (43.5; 95% CI, 42.7-44.2), and colon, rectum and anus (17.1, 95% CI, 16.6-17.6).

3.2 Cancer incidence in urban areas

A total of 388 917 women and 449 934 men were diagnosed with any cancer as recorded in the 22 registers in urban areas in China during the period 2008-2012. The ASRs of all cancers were 193.4 (95% CI, 192.7-194.0) per 100 000 person-years in women and 233.4 (95% CI, 232.7-234.1) per 100 000 person-years in men. The five most frequent cancers in women in urban areas were cancers of the breast (ASR 41.3 per 100 000 person-years; 95% CI, 41.0-41.6), lung (25.0; 95% CI, 24.8-25.2), colon and rectum (22.3; 95% CI, 22.1-22.5), uterus (18.1; 95% CI, 17.9-18.3), and thyroid (13.0; 95% CI, 12.8-13.2). In men, the highest ASRs were observed for lung cancer (52.8;

For peer review only - http://bmjopen.bmj.com/site/about/guidelines.xhtml

95% CI, 52.5-53.1), followed by cancer of colon and rectum (30.7; 95% CI, 30.4-30.9), liver and biliary passages (29.0; 95% CI, 28.7-29.2), stomach (23.0; 95% CI, 22.8-23.2), and prostate (13.0; 95% CI, 12.8-13.2).

3.3 Rate difference comparing rural and urban areas

Figure 1 presents the absolute difference in cancer incidence, i.e. RDs comparing rural and urban areas, in women and in men. The total cancer incidence was lower in women (RD -4.7 per 100 000 person-years; 95% CI, -6.4--3.1) but higher in men (39.9; 95% CI, 39.0-43.0) living in the rural areas than those in urban areas. Compared with those in urban areas, women living in rural areas had substantially higher incidence rates of cancers of the esophagus (RD 23.6 per 100 000 person-years; 95% CI, 23.1-24.2), stomach (16.0; 95% CI, 15.4-16.5), and liver and biliary passages cancer (8.0; 95% CI, 7.5-8.5), but lower incidence rates of cancers of the breast (-19.8; 95% CI, -20.4--19.2), thyroid (-9.6; 95% CI, -9.8--9.3), colon and rectum (-8.9; 95% CI, -9.3--8.4), ovary (-2.5; 95% CI, -2.7--2.2), and lung (-2.4; 95% CI, -3.0--1.8). Men residing in the rural areas had higher incidence rates of cancers of the stomach (RD 38.4 per 100 000 person-years; 95% CI, 37.4-39.3), esophagus (32.3; 95% CI, 31.6-33.1), and liver and biliary passages (16.8; 95% CI, 16.1-17.7), while those in urban areas had higher rates of cancers of colon and rectum (-13.6; 95% CI, -14.1--13.0), prostate cancer (-9.0; 95% CI, -9.3--8.7), and lip, oral cavity and pharynx (-5.1; 95% CI, -5.4--4.8), kidney (-4.1; 95% CI, -4.3--3.9), thyroid (-3.2; 95% CI, -3.3--3.0), and bladder (-2.6; 95% CI, -2.9--2.3) .

3.4 Rate ratio comparing rural and urban areas

The RRs measuring the urban-rural disparity cancer incidence on the relative scale are shown in **Figure 2**. The total cancer incidence rates were similar between women residing in rural areas and

For peer review only

those in urban areas as measured by RR (0.98; 95% CI, 0.97-0.98). However, the total cancer incidence rate in men in rural areas was around 20% higher than those in urban areas (RR 1.17; 95% CI, 1.16-1.18). When analyzed by cancer type, compared those in urban areas, women in rural areas had substantially higher incidence rates for cancers of the esophagus (RR 9.8; 95% CI, 9.4-10.1) and stomach (2.5; 95% CI, 2.5-2.6), but at least 50% lower incidence rates for cancers of urinary organs other than kidney and bladder (0.2; 95% CI, 0.2-0.3), thyroid cancer (0.3; 95% CI, 0.2-0.3), kidney cancer(0.5; 95% CI, 0.4-0.5), Hodgkin lymphoma (0.5; 95% CI, 0.3-0.7), and breast cancer (0.5; 95% CI, 0.5-0.5). Men in rural areas had higher incidence rates of cancers of the esophagus (RR 3.9; 95% CI, 3.8-4.0) and stomach (2.7; 95% CI, 2.6-2.7), but 50% or lower incidence rates of cancers of the thyroid (0.2; 95% CI, 0.2-0.3), kidney (0.3; 95% CI, 0.3-0.3), prostate (0.3; 95% CI, 0.3-0.3), mesothelioma (0.4; 95% CI, 0.3-0.6), testis (0.5; 95% CI, 0.4-0.6), Hodgkin lymphoma (0.5; 95% CI, 0.4-0.6), lip, oral cavity and pharynx (0.5; 95% CI, 0.5-0.5), and small intestine (0.5; 95% CI, 0.5-0.6).

4 DISCUSSION

The present study revealed substantial disparities in cancer incidence between rural and urban areas in China, and the patterns varied across cancer types and the sexes. The overall cancer incidence was higher in men living in rural areas compared with those in urban areas but showed less urban-rural disparity in women. Both men and women in rural areas had substantially higher incidence rates of cancers of the esophagus, stomach, and liver and biliary passages, while higher incidence rates were observed in urban areas for a number of other cancers, including cancers of colon and rectum, thyroid, breast, prostate, lip, oral cavity and pharynx, and kidney.

For peer review only - http://bmjopen.bmj.com/site/about/guidelines.xhtml

This study provided an updated assessment of the urban-rural disparity in cancer incidence in China on a nationwide scale. One major strength was that we used data from the 36 cancer registers of high quality in terms of completeness of coverage and accuracy, thereby lending validity to the findings. In addition, we comprehensively investigated urban-rural disparity for overall and site-specific cancer incidence by sex and on both absolute and relative scales. This study also has some limitations. First, the included registries only represented a limited proportion of the total population in China and were not in well balanced distribution regarding geographic regions, and thus, the strengths of the disparity measures may not exactly reflect the extent of nationwide urban-rural disparity in cancer incidence. Second, due to unavailability of complete data in all cancer registries, we were not able to analyse the data by histological type. Lastly but not least, the analyses were based on cancer incidence data only without information on risk factors, and we could not direct evaluate how the observed disparity could be explained by the corresponding risk factors.

Overall, our findings were consistent with previous reports. A study using data from 72 registries in the Chinese National Central Cancer Registry in 2009 showed that esophageal cancer was one of the most common cancers in China, and the incidence rate was particularly high in men in rural areas [3]. A more recent study including 177 cancer registries further confirmed the higher incidence and mortality rates of esophageal cancer in rural areas than in urban areas in both sexes 2011 [4]. In this study, we found that the incidence rate of esophageal cancer in women s was 9.8 times higher in rural arear than in urban areas, and men in rural areas also had 3.9 times higher risk of esophageal cancer than those in urban areas. Such striking urban-rural disparity may be partially explained the higher prevalence of major risk factors for esophageal cancer, including lower socioeconomic status, tobacco smoking, heavy alcohol use, and dietary factors, in rural areas

For peer review only - http://bmjopen.bmj.com/site/about/guidelines.xhtml

[14 15]. On the other hand, the etiology of esophageal cancer, particularly that of the main histological subtype esophageal squamous cell carcinoma in China, has not been fully elucidated [16]. There may be other mechanisms explaining the burden gap between rural and urban populations in China.

Higher incidence rates of stomach and liver cancers in rural areas than in urban areas in China have also been reported previously [5 6]. Our findings were in line with these reports, which stressed the need for targeted prevention strategies for these cancers in rural areas. As many modifiable factors, including obesity [17], nutrition [18], virus infection [19], alcohol use [20] and smoking [19] are important risk factors for stomach and/or liver cancers, primary prevention strategy targeting at these dimensions may be implemented in rural areas to narrow the urban-rural disparity in these cancers.

Thyroid and breast cancers are the most common cancer types in women, especially in the middle-aged group [21]. Higher incidence rates of thyroid and breast cancers in women in urban areas than in rural areas in China have been previously reported [7 8], which were in in line with our findings. Such urban-rural disparity might be contributable to the possible differences in prevalence of risk factors, e.g. smoking, obesity, radiation exposure, oral contraceptives usage and intake of vegetables [22-24], between women in rural and urban areas. Another potential explanation is the more frequent use of screening diagnostic procedures, such as ultrasound, Doppler examination, CT and MRI scanning, and biochemical markers, in urban residents, which might have increased the detection of these cancers [25]. The more frequent use of screening or diagnostic procedures, such as prostate-specific antigen testing, in urban residents may also partially explain the higher incidence rate of prostate cancer in men living in urban areas than in rural areas.

We found higher incidence rates of colorectal cancer in both men and women living in urban areas compared with their counterparts in rural areas, which were in agreement with some

For peer review only - http://bmjopen.bmj.com/site/about/guidelines.xhtml

other studies [5 26 27]. Such disparity might be explained by higher prevalence of certain risk factors, particularly those associated with Western lifestyles including low intake of fruit and vegetables, read meat consumption, and lack of physical activity, in those in urban areas. However, it was also likely due to differences in access to diagnostic and treatment services between urban and rural residents.–Nevertheless, considering the huge disease burden and possibility of early detection, both primary and secondary prevention strategies are highly needed for targeting urban populations, not only for minimizing the urban-rural disparity but also for reducing deaths from this cancer. A tool tick, comprehensively including lifestyle, obesity, and cardiometabolic factors may be used for prevention of colorectal cancer [28].

The etiology of some other cancers with notable urban-rural disparity, including oral cancer and kidney cancer, has not been well illustrated. A better management regarding tobacco smoking, alcohol use, sexually transmitted disease, dental hygiene, nutrition, and occupational hazards in urban residents might facilitate the reduction of disparity in the incidence of these cancers [29 30].

5 CONCLUSIONS

In summary, this updated assessment of the urban-rural disparity in cancer incidence in China revealed substantial urban-rural disparity which varies across cancer types and the sexes. Residents in rural areas had strikingly higher risk of upper gastrointestinal cancers than those in urban areas, while residents in urban areas had higher risk of some other cancers, mainly including colorectal cancer, thyroid cancer, female breast cancer, and prostate cancer. The observed urban-rural disparity may be explained by a combination of differential prevalence of risk factors and access to screening and diagnostic services. Cancer prevention strategies should be tailored for common cancers in rural and urban areas.

For peer review only - http://bmjopen.bmj.com/site/about/guidelines.xhtml

For peer review only

Additional information

Funding

This study was supported by the Swedish Cancer Society (grant number 190043) and Karolinska Institutet Research Foundation (grant number 2018-01558).

COMPETING INTERESTS

The authors declare that they have no [illegible]

Ethics approval and consent to participate

The analyses were solely based on publicly available data of population sizes and aggregate number of cancer cases and as such, ethics approval or consent to participate was not deemed to be necessary.

Author contribution

SHX designed the study, analyzed the data and reviewed the article. SY analyzed the data and drafted and reviewed the article. All authors read and approved the final manuscript.

Data availability statement

All data used are publicly available from the International Agency for Research on Cancer (IARC).

Conflict of interest

The authors declare no conflicts of interest.

For peer review only - http://bmjopen.bmj.com/site/about/guidelines.xhtml

References

1. Chen W, Xia C, Zheng R, et al. Disparities by province, age, and sex in site-specific cancer burden attributable to 23 potentially modifiable risk factors in China: a comparative risk assessment. *Lancet Glob Health* 2019;7(2):e257-e69. doi: 10.1016/s2214-109x(18)30488-1 [published Online First: 2019/01/27]
2. Pan R, Zhu M, Yu C, et al. Cancer incidence and mortality: A cohort study in China, 2008-2013. *Int J Cancer* 2017;141(7):1315-23. doi: 10.1002/ijc.30825 [published Online First: 2017/06/09]
3. Chen W, He Y, Zheng R, et al. Esophageal cancer incidence and mortality in China, 2009. *J Thorac Dis* 2013;5(1):19-26. doi: 10.3978/j.issn.2072-1439.2013.01.04 [published Online First: 2013/02/02]
4. Zeng H, Zheng R, Zhang S, et al. Esophageal cancer statistics in China, 2011: Estimates based on 177 cancer registries. *Thorac Cancer* 2016;7(2):232-7. doi: 10.1111/1759-7714.12322 [published Online First: 2016/04/05]
5. Yang L, Zheng R, Wang N, et al. Incidence and mortality of stomach cancer in China, 2014. *Chin J Cancer Res* 2018;30(3):291-98. doi: 10.21147/j.issn.1000-9604.2018.03.01 [published Online First: 2018/07/27]
6. Fei FR, Hu RY, Gong WW, et al. Analysis of Mortality and Survival Rate of Liver Cancer in Zhejiang Province in China: A General Population-Based Study. *Can J Gastroenterol Hepatol* 2019;2019:1074286. doi: 10.1155/2019/1074286 [published Online First: 2019/07/31]
7. Wen D, Wen X, Yang Y, et al. Urban rural disparity in female breast cancer incidence rate in China and the increasing trend in parallel with socioeconomic development and urbanization in a rural setting. *Thorac Cancer* 2018;9(2):262-72. doi: 10.1111/1759-7714.12575 [published Online First: 2017/12/28]
8. Yang L, Zheng R, Wang N, et al. [Analysis of incidence and mortality of thyroid cancer in China, 2010]. *Zhonghua Yu Fang Yi Xue Za Zhi* 2014;48(8):663-8. [published Online First: 2014/11/13]
9. CI5 XI - Home 2020 [Available from: https://ci5.iarc.fr/CI5-XI/Default.aspx.
10. Bray F CM, Mery L, Piñeros M, Znaor A, Zanetti R, Ferlay J. Cancer Incidence in Five Continents, Vol. XI (electronic version): Lyon: International Agency for Research on Cancer.; 2017 [Available from: https://ci5.iarc.fr.
11. O.B. Ahmad CB-P, A.D. Lopez, C.J.L. Murray, R. Lozano, M. Inoue. Age Standardization of Rates: A New WHO Standard. GPE Discussion Paper Series: No.31: World Health Organization, Geneva; 2001 [
12. Fay MP, Feuer EJ. Confidence intervals for directly standardized rates: a method based on the gamma distribution. *Stat Med* 1997;16(7):791-801.
13. Xie SH, Rabbani S, Petrick JL, et al. Racial and Ethnic Disparities in the Incidence of Esophageal Cancer in the United States, 1992-2013. *Am J Epidemiol* 2017;186(12):1341-51. doi: 10.1093/aje/kwx221 [published Online First: 2017/06/24]
14. Zhou X, Su Z, Deng H, et al. A comparative survey on alcohol and tobacco use in urban and rural populations in the Huaihua District of Hunan Province, China. *Alcohol* 2006;39(2):87-96. doi: 10.1016/j.alcohol.2006.07.003 [published Online First: 2006/12/01]
15. He YN, Zhai FY, Ge KY. [Measuring diet quality of chinese 18 - 59 years adult using chinese diet balance index]. *Wei Sheng Yan Jiu* 2005;34(4):442-4. [published Online First: 2005/10/19]
16. Xie SH, Lagergren J. Risk factors for oesophageal cancer. *Best Pract Res Clin Gastroenterol* 2018;36-37:3-8. doi: 10.1016/j.bpg.2018.11.008 [published Online First: 2018/12/16]
17. Larsson SC, Wolk A. Overweight, obesity and risk of liver cancer: a meta-analysis of cohort studies. *Br J Cancer* 2007;97(7):1005-8. doi: 10.1038/sj.bjc.6603932 [published Online First: 2007/08/19]

For peer review only

18. Yuan S, Carter P, Vithayathil M, et al. Iron Status and Cancer Risk in UK Biobank: A Two-Sample Mendelian Randomization Study. *Nutrients* 2020;12(2) doi: 10.3390/nu12020526 [published Online First: 2020/02/26]
19. Brenner H, Rothenbacher D, Arndt V. Epidemiology of stomach cancer. *Methods Mol Biol* 2009;472:467-77. doi: 10.1007/978-1-60327-492-0_23 [published Online First: 2008/12/25]
20. Bagnardi V, Rota M, Botteri E, et al. Alcohol consumption and site-specific cancer risk: a comprehensive dose-response meta-analysis. *Br J Cancer* 2015;112(3):580-93. doi: 10.1038/bjc.2014.579 [published Online First: 2014/11/26]
21. Yuan S, Kar S, Vithayathil M, et al. Causal associations of thyroid function and dysfunction with overall, breast and thyroid cancer: A two-sample Mendelian randomization study. *Int J Cancer* 2020 doi: 10.1002/ijc.32988 [published Online First: 2020/03/28]
22. Singletary SE. Rating the risk factors for breast cancer. *Ann Surg* 2003;237(4):474-82. doi: 10.1097/01.sla.0000059969.64262.87 [published Online First: 2003/04/05]
23. Sadeghi H, Rafei M, Bahrami M, et al. Attributable risk fraction of four lifestyle risk factors of thyroid cancer: a meta-analysis. *J Public Health (Oxf)* 2018;40(2):e91-e98. doi: 10.1093/pubmed/fdx088 [published Online First: 2017/10/05]
24. Dal Maso L, Bosetti C, La Vecchia C, et al. Risk factors for thyroid cancer: an epidemiological review focused on nutritional factors. *Cancer Causes Control* 2009;20(1):75-86. doi: 10.1007/s10552-008-9219-5 [published Online First: 2008/09/04]
25. Xie SH, Chen J, Zhang B, et al. Time trends and age-period-cohort analyses on incidence rates of thyroid cancer in Shanghai and Hong Kong. *BMC Cancer* 2014;14:975. doi: 10.1186/1471-2407-14-975 [published Online First: 2014/12/19]
26. Wen D, Zou W, Wen X, et al. Urban-rural disparity in colorectal cancer incidence and increasing trend in relation to socioeconomic development and urbanization in China. *J Int Med Res* 2018;46(10):4181-96. doi: 10.1177/0300060518791090 [published Online First: 2018/08/21]
27. Zhang SK, Zheng R, Chen Q, et al. Oral cancer incidence and mortality in China, 2011. *Chin J Cancer Res* 2015;27(1):44-51. doi: 10.3978/j.issn.1000-9604.2015.01.03 [published Online First: 2015/02/27]
28. Cornish AJ, Law PJ, Timofeeva M, et al. Modifiable pathways for colorectal cancer: a mendelian randomisation analysis. *Lancet Gastroenterol Hepatol* 2020;5(1):55-62. doi: 10.1016/s2468-1253(19)30294-8 [published Online First: 2019/11/02]
29. Ram H, Sarkar J, Kumar H, et al. Oral cancer: risk factors and molecular pathogenesis. *J Maxillofac Oral Surg* 2011;10(2):132-7. doi: 10.1007/s12663-011-0195-z [published Online First: 2012/06/02]
30. Scelo G, Larose TL. Epidemiology and Risk Factors for Kidney Cancer. *J Clin Oncol* 2018:Jco2018791905. doi: 10.1200/jco.2018.79.1905 [published Online First: 2018/10/30]

For peer review only

Table 1. Chinese cancer registries in the Cancer Incidence in Five Continents, XI volume

Region	Registry	Calendar period
Rural areas		
North	Cixian County	2008-2012
North	Shexian County	2008-2012
East	Qidong County	2008-2012
East	Jiashan County	2008-2012
East	Haimen County	2008-2012
East	Jianhu County	2010-2012
East	Guanyun	2008-2012
East	Sheyang	2008-2012
East	Xianju	2010-2012
Central	Linzhou County	2008-2012
Central	Hengdong	2009-2012
Central	Xiping	2010-2012
Central	Yanshi	2010-2012
Southwest	Yanting County	2008-2012
Urban areas		
North	Beijing	2008-2012
Northeast	Anshan City	2008-2012
Northeast	Harbin City, Nangang District	2008-2012
Northeast	Benxi	2008-2011
Northeast	Shenyang	2008-2012
East	Shanghai City	2008-2012
East	Hangzhou City	2008-2012
East	Jiaxing City	2008-2012
East	Hefei	2010-2012
East	Huaiyin District, Huai'an	2009-2012
East	Lianyungang	2008-2012
East	Maanshan	2008-2012
East	Tongling City	2008-2012
East	Wuxi	2010-2012
Central	Wuhan City	2008-2012
Central	Yueyanglou	2009-2012
South	Zhongshan City	2010-2012
South	Guangzhou	2010-2012
South	Hong Kong	2008-2012
South	Jiangmen	2010-2012
South	Liuzhou	2009-2012
South	Zhuhai	2010-2012

For peer review only

For peer review only - http://bmjopen.bmj.com/site/about/guidelines.xhtml

Table 2. Cancer incidence rates by anatomic site in women in China, 2008-2012

Cancer type	Rural areas			Urban areas		
	Number of cases	Crude Rate (95% CI)	Age-standardized rate (95% CI)	Number of cases	Crude Rate (95% CI)	Age-standardized rate (95% CI)
Lip, oral cavity and pharynx	850	3.7 (3.4, 3.9)	2.8 (2.6, 3.0)	8,329	6.2 (6.1, 6.3)	4.3 (4.2, 4.4)
Esophagus	8,408	36.2 (35.4, 36.9)	26.3 (25.8, 26.9)	6,034	4.5 (4.4, 4.6)	2.7 (2.6, 2.8)
Stomach	8,410	36.2 (35.4, 36.9)	26.5 (25.9, 27.1)	22,267	16.6 (16.4, 16.8)	10.5 (10.4, 10.7)
Small intestine	162	0.7 (0.6, 0.8)	0.5 (0.4, 0.6)	1,818	1.4 (1.3, 1.4)	0.9 (0.8, 0.9)
Colon, rectum and anus	4,268	18.4 (17.8, 18.9)	13.5 (13.1, 13.9)	47,907	35.7 (35.4, 36.1)	22.3 (22.1, 22.5)
Liver and biliary passages	6,084	26.2 (25.5, 26.8)	19.2 (18.8, 19.7)	24,268	18.1 (17.9, 18.3)	11.2 (11.1, 11.3)
Pancreas	1,555	6.7 (6.4, 7.0)	4.8 (4.6, 5.0)	10,067	7.5 (7.4, 7.7)	4.6 (4.5, 4.6)
Larynx	81	0.3 (0.3, 0.4)	0.3 (0.2, 0.3)	508	0.4 (0.3, 0.4)	0.2 (0.2, 0.3)
Lung	7,208	31.0 (30.3, 31.7)	22.6 (22.0, 23.1)	54,148	40.4 (40.1, 40.7)	25.0 (24.8, 25.2)
Bone	448	1.9 (1.8, 2.1)	1.6 (1.4, 1.7)	1,665	1.2 (1.2, 1.3)	1.0 (0.9, 1.0)
Melanoma of skin	109	0.5 (0.4, 0.6)	0.4 (0.3, 0.4)	905	0.7 (0.6, 0.7)	0.4 (0.4, 0.5)
Skin, excluding melanoma	563	2.4 (2.2, 2.6)	1.7 (1.6, 1.9)	5,353	4.0 (3.9, 4.1)	2.4 (2.4, 2.5)
Mesothelioma	31	0.1 (0.1, 0.2)	0.1 (0.1, 0.1)	319	0.2 (0.2, 0.3)	0.2 (0.1, 0.2)
Breast	6,539	28.1 (27.4, 28.8)	21.5 (21.0, 22.0)	80,535	60.1 (59.7, 60.5)	41.3 (41, 41.6)
Uterus	5,572	24 (23.3, 24.6)	18.4 (17.9, 18.8)	34,763	25.9 (25.7, 26.2)	18.1 (17.9, 18.3)
Ovary	1,269	5.5 (5.2, 5.8)	4.2 (4.0, 4.5)	12,782	9.5 (9.4, 9.7)	6.7 (6.6, 6.8)
Other female genital organs	200	0.9 (0.7, 1.0)	0.7 (0.6, 0.7)	2,038	1.5 (1.5, 1.6)	1.0 (1.0, 1.1)
Kidney	380	1.6 (1.5, 1.8)	1.3 (1.2, 1.4)	5,642	4.2 (4.1, 4.3)	2.8 (2.8, 2.9)
Bladder	507	2.2 (2.0, 2.4)	1.6 (1.5, 1.7)	5,029	3.8 (3.6, 3.9)	2.3 (2.2, 2.3)

For peer review only - http://bmjopen.bmj.com/site/about/guidelines.xhtml

Other urinary organs	73	0.3 (0.2, 0.4)	0.2 (0.2, 0.3)	2,271	1.7 (1.6, 1.8)	1.0 (1.0, 1.1)
Eye	51	0.2 (0.2, 0.3)	0.2 (0.1, 0.3)	217	0.2 (0.1, 0.2)	0.2 (0.2, 0.2)
Brain and nervous system	1,405	6.0 (5.7, 6.4)	4.9 (4.6, 5.1)	7,457	5.6 (5.4, 5.7)	4.2 (4.1, 4.3)
Thyroid	999	4.3 (4.0, 4.6)	3.4 (3.2, 3.7)	22,869	17.1 (16.8, 17.3)	13.0 (12.8, 13.2)
Other endocrine organs	165	0.7 (0.6, 0.8)	0.6 (0.5, 0.7)	1,229	0.9 (0.9, 1.0)	0.7 (0.7, 0.8)
Hodgkin lymphoma	42	0.2 (0.1, 0.2)	0.2 (0.1, 0.2)	508	0.4 (0.3, 0.4)	0.3 (0.3, 0.4)
Non-Hodgkin lymphoma	862	3.7 (3.5, 4.0)	2.9 (2.7, 3.1)	7,672	5.7 (5.6, 5.9)	3.9 (3.8, 4.0)
Multiple myeloma	185	0.8 (0.7, 0.9)	0.6 (0.5, 0.7)	2,162	1.6 (1.5, 1.7)	1.0 (1.0, 1.1)
Lymphoid leukemia	1,189	5.1 (4.8, 5.4)	4.6 (4.3, 4.8)	6,565	4.9 (4.8, 5.0)	4.2 (4.1, 4.3)
Other and unspecified	961	4.1 (3.9, 4.4)	3.2 (3.0, 3.4)	13,590	10.1 (10.0, 10.3)	6.7 (6.6, 6.8)
All sites but skin	58,013	249.5 (247.5, 251.5)	186.9 (185.4, 188.4)	383,564	286.2 (285.3, 287.1)	190.9 (190.3, 191.5)
All sites	58,576	251.9 (249.9, 253.9)	188.6 (187.1, 190.2)	388,917	290.2 (289.3, 291.1)	193.4 (192.7, 194)

For peer review only - http://bmjopen.bmj.com/site/about/guidelines.xhtml

Table 3. Cancer incidence rate by anatomic site in men in China, 2008-2012

Cancer type	Rural areas			Urban areas		
	Number of cases	Crude Rate (95% CI)	Age-standardized rate (95% CI)	Number of cases	Crude Rate (95% CI)	Age-standardized rate (95% CI)
Lip, oral cavity and pharynx	1,517	6.3 (6.0, 6.6)	5.0 (4.8, 5.3)	18,962	14.2 (14.0, 14.4)	10.1 (10.0, 10.2)
Esophagus	13,139	54.5 (53.6, 55.4)	43.5 (42.7, 44.2)	21,961	16.5 (16.2, 16.7)	11.1 (11.0, 11.3)
Stomach	18,515	76.8 (75.7, 77.9)	61.3 (60.4, 62.2)	45,179	33.9 (33.5, 34.2)	23.0 (22.8, 23.2)
Small intestine	190	0.8 (0.7, 0.9)	0.6 (0.5, 0.7)	2,238	1.7 (1.6, 1.7)	1.2 (1.1, 1.2)
Colon, rectum and anus	5,121	21.2 (20.7, 21.8)	17.1 (16.6, 17.6)	60,205	45.1 (44.8, 45.5)	30.7 (30.4, 30.9)
Liver and biliary passages	13,885	57.6 (56.7, 58.6)	45.8 (45.1, 46.6)	56,100	42.0 (41.7, 42.4)	29.0 (28.7, 29.2)
Pancreas	1,802	7.5 (7.1, 7.8)	6.0 (5.8, 6.3)	12,593	9.4 (9.3, 9.6)	6.4 (6.3, 6.5)
Larynx	522	2.2 (2.0, 2.4)	1.7 (1.6, 1.9)	5,679	4.3 (4.1, 4.4)	2.9 (2.8, 3.0)
Lung	15,740	65.3 (64.3, 66.3)	52.7 (51.9, 53.5)	104,382	78.2 (77.7, 78.7)	52.8 (52.5, 53.1)
Bone	612	2.5 (2.3, 2.7)	2.2 (2.0, 2.4)	2,041	1.5 (1.5, 1.6)	1.2 (1.2, 1.3)
Melanoma of skin	119	0.5 (0.4, 0.6)	0.4 (0.3, 0.5)	922	0.7 (0.6, 0.7)	0.5 (0.5, 0.5)
Skin, excluding melanoma	542	2.2 (2.1, 2.4)	1.9 (1.7, 2.0)	5,611	4.2 (4.1, 4.3)	2.9 (2.8, 3.0)
Mesothelioma	30	0.1 (0.1, 0.2)	0.1 (0.1, 0.1)	477	0.4 (0.3, 0.4)	0.2 (0.2, 0.3)
Breast	68	0.3 (0.2, 0.4)	0.2 (0.2, 0.3)	677	0.5 (0.5, 0.5)	0.4 (0.3, 0.4)
Prostate	1,152	4.8 (4.5, 5.1)	4.0 (3.8, 4.3)	25,963	19.5 (19.2, 19.7)	13.0 (12.8, 13.2)
Testis	103	0.4 (0.3, 0.5)	0.4 (0.3, 0.5)	1,176	0.9 (0.8, 0.9)	0.8 (0.8, 0.9)
Other male genital organs	184	0.8 (0.7, 0.9)	0.6 (0.5, 0.7)	1,371	1.0 (1.0, 1.1)	0.7 (0.7, 0.8)
Kidney	535	2.2 (2.0, 2.4)	1.8 (1.7, 2.0)	11,201	8.4 (8.2, 8.6)	5.9 (5.8, 6.0)
Bladder	1,610	6.7 (6.4, 7.0)	5.5 (5.2, 5.7)	16,013	12.0 (11.8, 12.2)	8.1 (8.0, 8.2)

For peer review only

Other urinary organs	121	0.5 (0.4, 0.6)	0.4 (0.3, 0.5)	2,449	1.8 (1.8, 1.9)	1.2 (1.2, 1.3)
Eye	49	0.2 (0.2, 0.3)	0.2 (0.1, 0.3)	236	0.2 (0.2, 0.2)	0.2 (0.2, 0.2)
Brain and nervous system	1,496	6.2 (5.9, 6.5)	5.3 (5.0, 5.5)	7,454	5.6 (5.5, 5.7)	4.4 (4.3, 4.5)
Thyroid	293	1.2 (1.1, 1.4)	1.0 (0.9, 1.1)	7,193	5.4 (5.3, 5.5)	4.2 (4.1, 4.3)
Other endocrine organs	130	0.5 (0.5, 0.6)	0.5 (0.4, 0.5)	1,221	0.9 (0.9, 1.0)	0.8 (0.7, 0.8)
Hodgkin lymphoma	63	0.3 (0.2, 0.3)	0.2 (0.2, 0.3)	740	0.6 (0.5, 0.6)	0.5 (0.4, 0.5)
Non-Hodgkin lymphoma	1,266	5.3 (5.0, 5.5)	4.3 (4.1, 4.6)	10,025	7.5 (7.4, 7.7)	5.5 (5.4, 5.6)
Multiple myeloma	286	1.2 (1.1, 1.3)	0.9 (0.8, 1.1)	3,096	2.3 (2.2, 2.4)	1.6 (1.5, 1.6)
Lymphoid leukemia	1,433	5.9 (5.6, 6.3)	5.4 (5.1, 5.7)	8,751	6.6 (6.4, 6.7)	5.8 (5.6, 5.9)
Other and unspecified	1,186	4.9 (4.6, 5.2)	4.1 (3.9, 4.3)	16,018	12.0 (11.8, 12.2)	8.5 (8.4, 8.7)
All sites but skin	81,167	336.7 (334.4, 339.1)	271.5 (269.6, 273.4)	444,323	333 (332, 333.9)	230.5 (229.8, 231.2)
All sites	81,709	339.0 (336.7, 341.3)	273.4 (271.5, 275.2)	449,934	337.2 (336.2, 338.1)	233.4 (232.7, 234.1)

For peer review only

For peer review only - http://bmjopen.bmj.com/site/about/guidelines.xhtml

Figure legends

Figure 1. Rate differences and 95% confidence intervals in cancer incidence comparing women (A) and men (B) in rural versus urban areas in China, 2008-2012

Figure 2. Rate ratios and 95% confidence intervals in cancer incidence comparing women (A) and men (B) in rural versus urban areas in China, 2008-2012

For peer review only

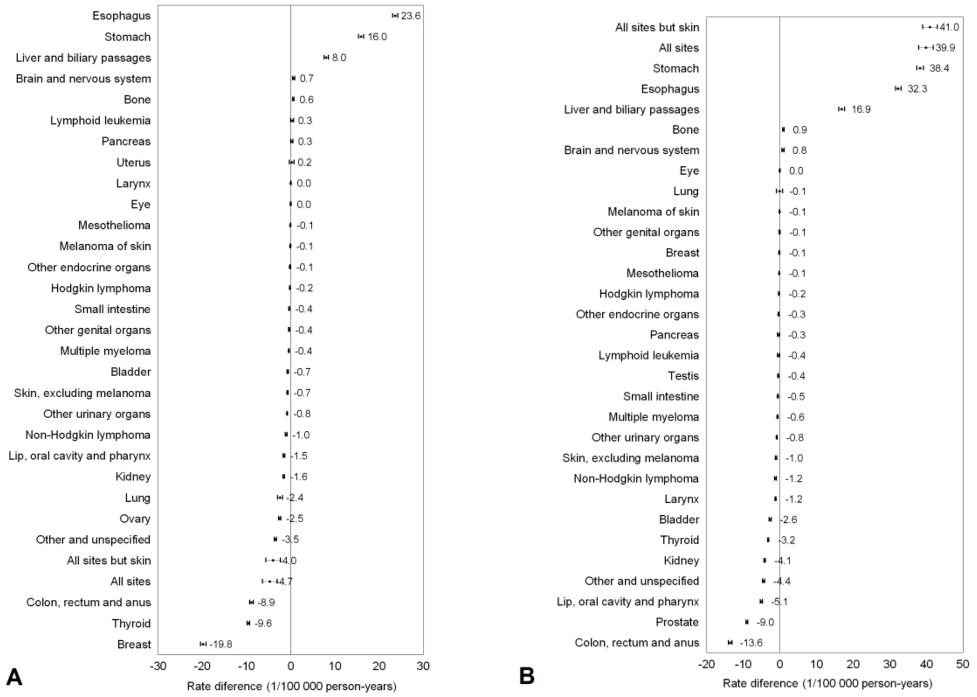

Figure 1. Rate differences and 95% confidence intervals in cancer incidence comparing women (A) and men (B) in rural versus urban areas in China, 2008-2012

721x524mm (96 x 96 DPI)

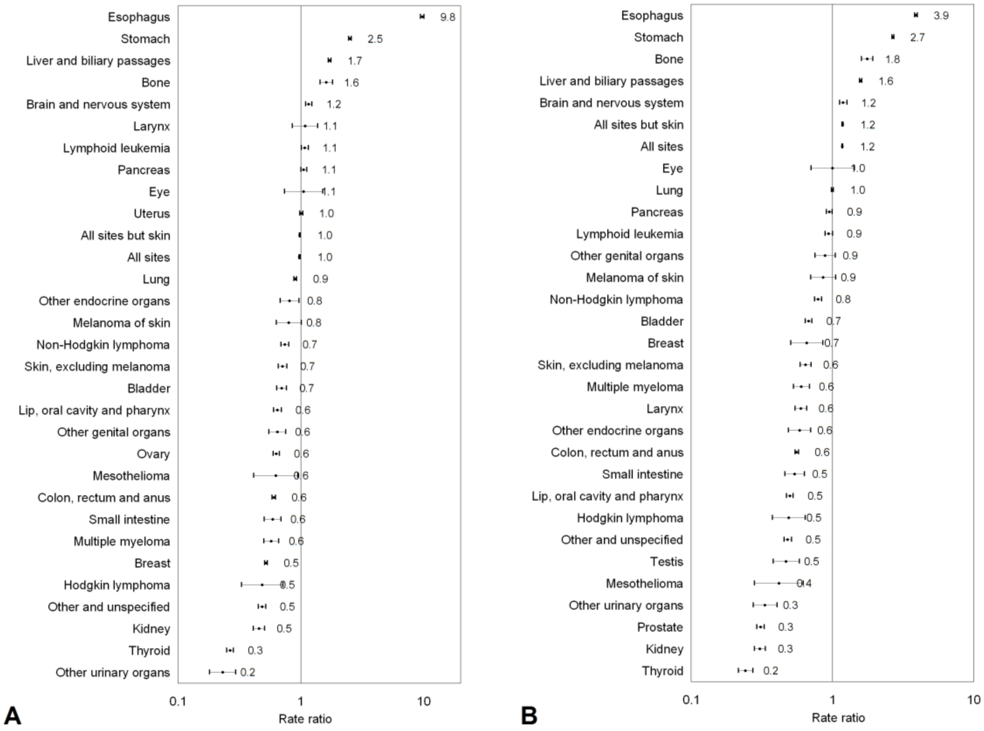

Figure 2. Rate ratios and 95% confidence intervals in cancer incidence comparing women (A) and men (B) in rural versus urban areas in China, 2008-2012

677x514mm (96 x 96 DPI)

STROBE 2007 (v4) Statement—Checklist of items that should be included in reports of *cross-sectional studies*

Section/Topic	Item #	Recommendation	Reported on page #
**Title and abstract**	1	(*a*) Indicate the study's design with a commonly used term in the title or the abstract	1
		(*b*) Provide in the abstract an informative and balanced summary of what was done and what was found	2
**Introduction**			
Background/rationale	2	Explain the scientific background and rationale for the investigation being reported	4
Objectives	3	State specific objectives, including any prespecified hypotheses	4
**Methods**			
Study design	4	Present key elements of study design early in the paper	5
Setting	5	Describe the setting, locations, and relevant dates, including periods of recruitment, exposure, follow-up, and data collection	5
Participants	6	(*a*) Give the eligibility criteria, and the sources and methods of selection of participants	5
Variables	7	Clearly define all outcomes, exposures, predictors, potential confounders, and effect modifiers. Give diagnostic criteria, if applicable	5
Data sources/ measurement	8*	For each variable of interest, give sources of data and details of methods of assessment (measurement). Describe comparability of assessment methods if there is more than one group	5
Bias	9	Describe any efforts to address potential sources of bias	*5*
Study size	10	Explain how the study size was arrived at	5
Quantitative variables	11	Explain how quantitative variables were handled in the analyses. If applicable, describe which groupings were chosen and why	5
Statistical methods	12	(*a*) Describe all statistical methods, including those used to control for confounding	5
		(*b*) Describe any methods used to examine subgroups and interactions	n/a
		(*c*) Explain how missing data were addressed	n/a
		(*d*) If applicable, describe analytical methods taking account of sampling strategy	n/a
		(*e*) Describe any sensitivity analyses	n/a
**Results**			

For peer review only

Participants	13*	(a) Report numbers of individuals at each stage of study—eg numbers potentially eligible, examined for eligibility, confirmed eligible, included in the study, completing follow-up, and analysed	6
		(b) Give reasons for non-participation at each stage	n/a
		(c) Consider use of a flow diagram	n/a
Descriptive data	14*	(a) Give characteristics of study participants (eg demographic, clinical, social) and information on exposures and potential confounders	6-8
		(b) Indicate number of participants with missing data for each variable of interest	n/a
Outcome data	15*	Report numbers of outcome events or summary measures	6-8
Main results	16	(*a*) Give unadjusted estimates and, if applicable, confounder-adjusted estimates and their precision (eg, 95% confidence interval). Make clear which confounders were adjusted for and why they were included	6-8
		(*b*) Report category boundaries when continuous variables were categorized	
		(*c*) If relevant, consider translating estimates of relative risk into absolute risk for a meaningful time period	n/a
Other analyses	17	Report other analyses done—eg analyses of subgroups and interactions, and sensitivity analyses	n/a
**Discussion**			
Key results	18	Summarise key results with reference to study objectives	8
Limitations	19	Discuss limitations of the study, taking into account sources of potential bias or imprecision. Discuss both direction and magnitude of any potential bias	8-9
Interpretation	20	Give a cautious overall interpretation of results considering objectives, limitations, multiplicity of analyses, results from similar studies, and other relevant evidence	8-9
Generalisability	21	Discuss the generalisability (external validity) of the study results	8-9
**Other information**			
Funding	22	Give the source of funding and the role of the funders for the present study and, if applicable, for the original study on which the present article is based	12

*Give information separately for cases and controls in case-control studies and, if applicable, for exposed and unexposed groups in cohort and cross-sectional studies.

Note: An Explanation and Elaboration article discusses each checklist item and gives methodological background and published examples of transparent reporting. The STROBE checklist is best used in conjunction with this article (freely available on the Web sites of PLoS Medicine at http://www.plosmedicine.org/, Annals of Internal Medicine at http://www.annals.org/, and Epidemiology at http://www.epidem.com/). Information on the STROBE Initiative is available at www.strobe-statement.org.

For peer review only

BMJ Open

Urban-rural disparity in cancer incidence in China, 2008-2012: A cross-sectional analysis of data from 36 cancer registers

Journal:	*BMJ Open*
Manuscript ID	bmjopen-2020-042762.R1
Article Type:	Original research
Date Submitted by the Author:	26-Jan-2021
Complete List of Authors:	Yuan, Shuai; Karolinska Institutet, Institute of Environmental Medicine<br>Xie, Shao-Hua; Karolinska University Hospital, Department of Molecular Medicine and Surgery
**Primary Subject Heading**:	Epidemiology
Secondary Subject Heading:	Oncology, Public health
Keywords:	EPIDEMIOLOGY, ONCOLOGY, PUBLIC HEALTH

SCHOLARONE™
Manuscripts

I, the Submitting Author has the right to grant and does grant on behalf of all authors of the Work (as defined in the below author licence), an exclusive licence and/or a non-exclusive licence for contributions from authors who are: i) UK Crown employees; ii) where BMJ has agreed a CC-BY licence shall apply, and/or iii) in accordance with the terms applicable for US Federal Government officers or employees acting as part of their official duties; on a worldwide, perpetual, irrevocable, royalty-free basis to BMJ Publishing Group Ltd ("BMJ") its licensees and where the relevant Journal is co-owned by BMJ to the co-owners of the Journal, to publish the Work in this journal and any other BMJ products and to exploit all rights, as set out in our licence.

The Submitting Author accepts and understands that any supply made under these terms is made by BMJ to the Submitting Author unless you are acting as an employee on behalf of your employer or a postgraduate student of an affiliated institution which is paying any applicable article publishing charge ("APC") for Open Access articles. Where the Submitting Author wishes to make the Work available on an Open Access basis (and intends to pay the relevant APC), the terms of reuse of such Open Access shall be governed by a Creative Commons licence – details of these licences and which Creative Commons licence will apply to this Work are set out in our licence referred to above.

Other than as permitted in any relevant BMJ Author's Self Archiving Policies, I confirm this Work has not been accepted for publication elsewhere, is not being considered for publication elsewhere and does not duplicate material already published. I confirm all authors consent to publication of this Work and authorise the granting of this licence.

For peer review only

Urban-rural disparity in cancer incidence in China, 2008-2012: A cross-sectional analysis of data from 36 cancer registers

Running head: Cancer disparity in China

Shuai Yuan, MB, MSc[1], Shao-Hua Xie, MD, PhD[2*]

Affiliations

[1] Unit of Cardiovascular and Nutritional Epidemiology, Institute of Environmental Medicine, Karolinska Institutet, Stockholm, Sweden.

[2] Upper Gastrointestinal Surgery, Department of Molecular Medicine and Surgery, Karolinska Institutet, Karolinska University Hospital, Stockholm, Sweden

[*]Correspondence

Shao-Hua Xie, MD, PhD, Upper Gastrointestinal Surgery, Department of Molecular medicine and Surgery, Karolinska Institutet, Karolinska University Hospital, Retzius väg 13a, 4th Floor, 17 177 Stockholm, Sweden. Email: shaohua.xie@ki.se

Words of Abstract / Manuscript: 224 / 2286

Number of tables / figures: 2 / 2

For peer review only

Abstract

Objective: The substantial differences in socioeconomic and lifestyle exposures between urban and rural areas in China may lead to urban-rural disparity in cancer risk. This study aimed to assess the urban-rural disparity in cancer incidence in China.

Methods: Using data from 36 regional cancer registries in China in 2008-2012, we compared the age-standardized incidence rates of cancer by sex and anatomic site between rural and urban areas. We calculated the rate difference and rate ratio comparing rates in rural versus urban areas by sex and cancer type.

Results The incidence rate of all cancers in women was slightly lower in rural areas than in urban areas, but the total cancer rate in men was higher in rural areas than in urban areas. The incidence rates in women were higher in rural areas than in urban areas for cancers of the esophagus, stomach, and liver and biliary passages, but lower for cancers of thyroid and breast. Men residing in rural areas had higher incidence rates for cancers of the esophagus, stomach, and liver and biliary passages, but lower rates for prostate cancer, lip, oral cavity and pharynx cancer, and colorectal cancer.

Conclusions: Our findings suggest substantial urban-rural disparity in cancer incidence in China, which varies across cancer types and the sexes. Cancer prevention strategies should be tailored for common cancers in rural and urban areas.

Keywords: Neoplasms; incidence; socioeconomic status; spatial distribution; epidemiology

For peer review only

For peer review only - http://bmjopen.bmj.com/site/about/guidelines.xhtml

Strengths and limitations of this study

- We used data from the 36 cancer registers of high quality to investigate urban-rural disparity for overall and site-specific cancer incidence by sex and on both absolute and relative scales.
- The cross-sectional design limited the causal inference in geographical disparities to cancer incidence.
- Included registries only represented a limited proportion of the total population in China and were not in well balanced distribution regarding geographic regions.
- We were not able to analyze the data by histological type.
- We could not evaluate the associations of cancer disparity with gross domestic product, human development index or corresponding risk factors.

For peer review only

Introduction

Cancer is one of the leading causes of death worldwide, including in China. According to China Health Statistics Yearbook, an estimated 2.2 million cancer deaths occurred in China in 2017, accounting for around 25% of all deaths in this year [1]. Moreover, the past few decades have witnessed rapid urbanization and industrialization in China, and the uneven distribution of wealth and lifestyle profiles across regions in the nation during this process may have in turn resulted in substantial urban-rural disparity in cancer risk. Previous studies have suggested marked urban-rural disparity in cancer incidence in China, and such disparity seems varying across cancer types [2-8]. However, most of these previous studies were limited in a certain area or province, or specific types of cancer only. Due to historically limited population-based cancer registry data in China, the urban-rural disparity in cancer incidence has not been well characterized.

China has recently established the National Central Cancer Registry and data from 36 regional cancer registries have been included in the latest release of *Cancer Incidence in Five Continents* series published by the International Agency for Research on Cancer [9]. This provides a new opportunity to assess the regional disparity in cancer incidence in China on a national scale. Therefore, using data from these high-quality registries, we conducted the present study to characterize the urban-rural disparity in total and type-specific cancer incidence by sex in China in 2008-2012.

Methods

Data source

We extracted data on cancer incidence and population sizes from 36 regional cancer registries, including 14 registries in rural areas and 22 in urban areas, in China during 2008-2012 which are

For peer review only - http://bmjopen.bmj.com/site/about/guidelines.xhtml

included in the XI volume of *Cancer Incidence in Five Continents* series published by the International Agency for Research on Cancer [10]. These registries are located in different geographical regions throughout the country. Cancer cases were defined by International Classification of Disease codes, which are listed in a webpage of the International Agency for Research on Cancer (https://ci5.iarc.fr/CI5-XI/PDF/Chapter%203.pdf). Detailed information on the included registries is presented in **Supplementary Table 1 and Table 2**. The case-weighted means of percentage of case defined by death certificate only were ~1.9 in male, ~1.5 in female and ~1.7 in both male and female among included registries. Included population from 36 cancer registries made up ~5% of the total population in China. We pooled the numbers of cases and population sizes at risk from multiple registers separately for urban and rural areas by sex and cancer type (anatomic site).

Statistical analysis

We calculated the crude and age-standardized incidence rates (ASRs) and their 95% confidence intervals (CIs) by sex and cancer type separately for rural and urban areas. The ASRs were computed by using the direct standardization method with World Standard Population 2000 as reference [11]. The 95% CIs of crude rates were estimated under the assumption of Poisson distribution and CIs for ASRs were calculated based on the gamma distribution assuming that the standardized rate is a weighted sum of independent Poisson random variables [12]. The urban-rural disparities in cancer incidence were quantitatively assessed with two disparity measures (i.e., rate difference (RD) on the absolute scale and rate ratio (RR) on the relative scale) [13]. We calculated the RD (ASR in rural areas – ASR in urban areas) and rate ratio (ratio of ASRs in rural areas

For peer review only - http://bmjopen.bmj.com/site/about/guidelines.xhtml

relative to urban areas) with 95% CIs by sex and cancer type. All statistical analyses were performed using the SAS version 9.4 (SAS Institute, Cary, NC) and two-sided.

Patient and Public Involvement

The analyses were solely based on publicly available data of population sizes and aggregated number of cancer cases and as such, ethics approval or consent to participate was not deemed to be necessary. No individual-level data were involved in the study or in defining the research question or outcome measures.

Results

Cancer incidence in rural areas

The crude rates and ASRs in rural and urban areas are displayed in **Table 1** for women and in **Table 2** for men. In total, 58,576 female and 81,709 male new cancer cases were recorded in 14 cancer registers in rural areas in China during the period 2008-2012, contributing to the ASRs of 188.6 (95% CI, 187.1, 190.2) per 100,000 population per year in women and 273.4 (95% CI, 271.5, 275.2) per 100,000 population per year in men. In women residing in rural areas, the ASRs were observed for stomach cancer, followed by cancers of the esophagus, lung, breast, and liver and biliary passages. In men in rural areas, stomach cancer was also the most common cancer type, followed by cancers of the lung, liver and biliary passages, esophagus, and colon, rectum and anus.

Cancer incidence in urban areas

A total of 388,917 women and 449,934 men were diagnosed with any cancer as recorded in the 22 registers in urban areas in China during the period 2008-2012. The ASRs of all cancers were 193.4

For peer review only - http://bmjopen.bmj.com/site/about/guidelines.xhtml

(95% CI, 192.7, 194.0) per 100,000 population per year in women and 233.4 (95% CI, 232.7, 234.1) per 100,000 population per year in men. The five most frequent cancers in women in urban areas were cancers of the breast, lung, colon and rectum, uterus, and thyroid. In men, the highest ASRs were observed for lung cancer, followed by cancer of colon and rectum, liver and biliary passages, stomach, and prostate.

Rate difference comparing rural and urban areas

Figure 1 presents the absolute difference in cancer incidence, i.e., RDs comparing rural and urban areas, in women and in men. The total cancer incidence was lower in women (RD, -4.7 per 100,000 population per year, 95% CI, -6.4, -3.1) but higher in men (RD, 39.9, 95% CI, 39.0, 43.0) living in the rural areas than those in urban areas. Compared with those in urban areas, women living in rural areas had substantially higher incidence rates of cancers of the esophagus, stomach, and liver and biliary passages cancer, but lower incidence rates of cancers of the breast, thyroid, colon and rectum, ovary, and lung. Men residing in the rural areas had higher incidence rates of cancers of the stomach, esophagus, and liver and biliary passages, while those in urban areas had higher rates of cancers of colon and rectum, prostate cancer, and lip, oral cavity and pharynx, kidney, thyroid, and bladder.

Rate ratio comparing rural and urban areas

The RRs measuring the urban-rural disparity cancer incidence on the relative scale are shown in **Figure 2**. The total cancer incidence rates were similar between women residing in rural areas and those in urban areas as measured by RR (0.98, 95% CI, 0.97, 0.98). However, the total cancer incidence rate in men in rural areas was around 20% higher than those in urban areas (1.17, 95%

For peer review only - http://bmjopen.bmj.com/site/about/guidelines.xhtml

CI, 1.16, 1.18). When analyzed by cancer type, compared those in urban areas, women in rural areas had substantially higher incidence rates for cancers of the esophagus and stomach, but at least 50% lower incidence rates for cancers of urinary organs other than kidney and bladder, thyroid cancer, kidney cancer, Hodgkin lymphoma, and breast cancer. Men in rural areas had higher incidence rates of cancers of the esophagus and stomach, but 50% or lower incidence rates of cancers of the thyroid, kidney, prostate, mesothelioma, testis, Hodgkin lymphoma, lip, oral cavity and pharynx, and small intestine.

Discussion

The present study revealed substantial disparities in cancer incidence between rural and urban areas in China, and the patterns varied across cancer types and the sexes. The overall cancer incidence was higher in men living in rural areas compared with those in urban areas but showed less urban-rural disparity in women. Both men and women in rural areas had substantially higher incidence rates of cancers of the esophagus, stomach, and liver and biliary passages, while higher incidence rates were observed in urban areas for a number of other cancers, including cancers of colon and rectum, thyroid, breast, prostate, lip, oral cavity and pharynx, and kidney. This study provided an updated assessment of the urban-rural disparity in cancer incidence in China on a nationwide scale.

Overall, our findings were consistent with previous reports. A study using data from 72 registries in the Chinese National Central Cancer Registry in 2009 showed that esophageal cancer was one of the most common cancers in China, and the incidence rate was particularly high in men in rural areas [3]. A more recent study including 177 cancer registries further confirmed the higher incidence and mortality rates of esophageal cancer in rural areas than in urban areas in both sexes

For peer review only - http://bmjopen.bmj.com/site/about/guidelines.xhtml

2011 [4]. In this study, we found that the incidence rate of esophageal cancer in women s was 9.8 times higher in rural arear than in urban areas, and men in rural areas also had 3.9 times higher risk of esophageal cancer than those in urban areas. Such striking urban-rural disparity may be partially explained the higher prevalence of major risk factors for esophageal cancer, including lower socioeconomic status, tobacco smoking, heavy alcohol use, and dietary factors, in rural areas [14 15]. On the other hand, the etiology of esophageal cancer, particularly that of the main histological subtype esophageal squamous cell carcinoma in China, has not been fully elucidated [16]. There may be other mechanisms explaining the burden gap between rural and urban populations in China.

Higher incidence rates of stomach and liver cancers in rural areas than in urban areas in China have also been reported previously [5 6]. Our findings were in line with these reports, which stressed the need for targeted prevention strategies for these cancers in rural areas. As many modifiable factors, including obesity [17], nutrition [18], Helicobacter pylori infection [19], virus infection [20], alcohol use [21] and smoking [20] are important risk factors for stomach and/or liver cancers, primary prevention strategy targeting at these dimensions may be implemented in rural areas to narrow the urban-rural disparity in these cancers.

Thyroid and breast cancers are the most common cancer types in women, especially in the middle-aged group [22]. Higher incidence rates of thyroid and breast cancers in women in urban areas than in rural areas in China have been previously reported [7 8], which were in in line with our findings. Such urban-rural disparity might be contributable to the possible differences in prevalence of risk factors, e.g., smoking, obesity, radiation exposure, oral contraceptives usage and intake of vegetables [23-25], between women in rural and urban areas. Another potential explanation is the more frequent use of screening diagnostic procedures, such as ultrasound, Doppler examination, CT and MRI scanning, and biochemical markers, in urban residents, which

For peer review only - http://bmjopen.bmj.com/site/about/guidelines.xhtml

might have increased the detection of these cancers at an early stage, and thus, led to somewhat overestimated incidence of these cancers in recent years [26]. The more frequent use of screening or diagnostic procedures, such as prostate-specific antigen testing, in urban residents may also partially explain the higher incidence rate of prostate cancer in men living in urban areas than in rural areas.

We found higher incidence rates of colorectal cancer in both men and women living in urban areas compared with their counterparts in rural areas, which were in agreement with some other studies [5 27 28]. Such disparity might be explained by higher prevalence of certain risk factors, particularly those associated with Western lifestyles including low intake of fruit and vegetables, read meat consumption, and lack of physical activity, in those in urban areas. However, it was also likely due to differences in access to diagnostic and treatment services between urban and rural residents. Nevertheless, considering the huge disease burden and possibility of early detection, both primary and secondary prevention strategies are highly needed for targeting urban populations, not only for minimizing the urban-rural disparity but also for reducing deaths from this cancer. A tool tick, comprehensively including lifestyle, obesity, and cardiometabolic factors may be used for prevention of colorectal cancer [29].

The etiology of some other cancers with notable urban-rural disparity, including oral cancer and kidney cancer, has not been well illustrated. A better management regarding tobacco smoking, alcohol use, sexually transmitted disease, dental hygiene, nutrition, and occupational hazards in urban residents might facilitate the reduction of disparity in the incidence of these cancers [30 31].

There are strengths of the present study. One major strength was that we used data from the 36 cancer registers of high quality in terms of completeness of coverage and accuracy, thereby

For peer review only - http://bmjopen.bmj.com/site/about/guidelines.xhtml

lending validity to the findings. In addition, we comprehensively investigated urban-rural disparity for overall and site-specific cancer incidence by sex and on both absolute and relative scales.

This study also has some limitations. This study was embedded in the cross-sectional design, which might not conclude the causation between geographical disparities to cancer incidence. In addition, we used data constructed at the city-level. Thus, whether our findings could be generalized to the individuals needs verification. The included registries only represented a limited proportion of the total population in China and were not in well balanced distribution regarding geographic regions, and thus, the strengths of the disparity measures may not exactly reflect the extent of nationwide urban-rural disparity in cancer incidence. Due to unavailability of complete data in all cancer registries, we were not able to analyze the data by histological type, or the associations of gross domestic product or human development index with ASRs. Last but not least, the analyses were based on cancer incidence data only without information on risk factors, and we could not direct evaluate how the observed disparity could be explained by the corresponding risk factors.

Conclusions

This updated assessment of the urban-rural disparity in cancer incidence in China revealed substantial urban-rural disparity which varies across cancer types and the sexes. The observed urban-rural disparity may be explained by a combination of differential prevalence of risk factors and access to screening and diagnostic services. Cancer prevention strategies should be tailored for common cancers in rural and urban areas.

For peer review only - http://bmjopen.bmj.com/site/about/guidelines.xhtml

Additional information

Acknowledgements: We would like to thank International Agency for Research on Cancer for sharing data.

Contributors: SHX designed the study, analyzed the data and reviewed the article. SY analyzed the data and drafted and reviewed the article. All authors read and approved the final manuscript.

Funding: This study was supported by the Swedish Cancer Society (grant number 190043) and Karolinska Institutet Research Foundation (grant number 2018-01558).

Competing interests: None declared.

Patient consent for publication: Not required.

Data availability statement: All data used are publicly available from the International Agency for Research on Cancer.

For peer review only

References

1. Chen W, Xia C, Zheng R, et al. Disparities by province, age, and sex in site-specific cancer burden attributable to 23 potentially modifiable risk factors in China: a comparative risk assessment. *Lancet Glob Health* 2019;7(2):e257-e69. doi: 10.1016/s2214-109x(18)30488-1 [published Online First: 2019/01/27]
2. Pan R, Zhu M, Yu C, et al. Cancer incidence and mortality: A cohort study in China, 2008-2013. *Int J Cancer* 2017;141(7):1315-23. doi: 10.1002/ijc.30825 [published Online First: 2017/06/09]
3. Chen W, He Y, Zheng R, et al. Esophageal cancer incidence and mortality in China, 2009. *J Thorac Dis* 2013;5(1):19-26. doi: 10.3978/j.issn.2072-1439.2013.01.04 [published Online First: 2013/02/02]
4. Zeng H, Zheng R, Zhang S, et al. Esophageal cancer statistics in China, 2011: Estimates based on 177 cancer registries. *Thorac Cancer* 2016;7(2):232-7. doi: 10.1111/1759-7714.12322 [published Online First: 2016/04/05]
5. Yang L, Zheng R, Wang N, et al. Incidence and mortality of stomach cancer in China, 2014. *Chin J Cancer Res* 2018;30(3):291-98. doi: 10.21147/j.issn.1000-9604.2018.03.01 [published Online First: 2018/07/27]
6. Fei FR, Hu RY, Gong WW, et al. Analysis of Mortality and Survival Rate of Liver Cancer in Zhejiang Province in China: A General Population-Based Study. *Can J Gastroenterol Hepatol* 2019;2019:1074286. doi: 10.1155/2019/1074286 [published Online First: 2019/07/31]
7. Wen D, Wen X, Yang Y, et al. Urban rural disparity in female breast cancer incidence rate in China and the increasing trend in parallel with socioeconomic development and urbanization in a rural setting. *Thorac Cancer* 2018;9(2):262-72. doi: 10.1111/1759-7714.12575 [published Online First: 2017/12/28]
8. Yang L, Zheng R, Wang N, et al. [Analysis of incidence and mortality of thyroid cancer in China, 2010]. *Zhonghua Yu Fang Yi Xue Za Zhi* 2014;48(8):663-8. [published Online First: 2014/11/13]
9. CI5 XI - Home 2020 [Available from: https://ci5.iarc.fr/CI5-XI/Default.aspx.
10. Bray F CM, Mery L, Piñeros M, Znaor A, Zanetti R, Ferlay J. Cancer Incidence in Five Continents, Vol. XI (electronic version): Lyon: International Agency for Research on Cancer.; 2017 [Available from: https://ci5.iarc.fr.
11. O.B. Ahmad CB-P, A.D. Lopez, C.J.L. Murray, R. Lozano, M. Inoue. Age Standardization of Rates: A New WHO Standard. GPE Discussion Paper Series: No.31: World Health Organization, Geneva; 2001 [
12. Fay MP, Feuer EJ. Confidence intervals for directly standardized rates: a method based on the gamma distribution. *Stat Med* 1997;16(7):791-801.
13. Xie SH, Rabbani S, Petrick JL, et al. Racial and Ethnic Disparities in the Incidence of Esophageal Cancer in the United States, 1992-2013. *Am J Epidemiol* 2017;186(12):1341-51. doi: 10.1093/aje/kwx221 [published Online First: 2017/06/24]
14. Zhou X, Su Z, Deng H, et al. A comparative survey on alcohol and tobacco use in urban and rural populations in the Huaihua District of Hunan Province, China. *Alcohol* 2006;39(2):87-96. doi: 10.1016/j.alcohol.2006.07.003 [published Online First: 2006/12/01]
15. He YN, Zhai FY, Ge KY. [Measuring diet quality of chinese 18 - 59 years adult using chinese diet balance index]. *Wei Sheng Yan Jiu* 2005;34(4):442-4. [published Online First: 2005/10/19]
16. Xie SH, Lagergren J. Risk factors for oesophageal cancer. *Best Pract Res Clin Gastroenterol* 2018;36-37:3-8. doi: 10.1016/j.bpg.2018.11.008 [published Online First: 2018/12/16]
17. Larsson SC, Wolk A. Overweight, obesity and risk of liver cancer: a meta-analysis of cohort studies. *Br J Cancer* 2007;97(7):1005-8. doi: 10.1038/sj.bjc.6603932 [published Online First: 2007/08/19]

For peer review only - http://bmjopen.bmj.com/site/about/guidelines.xhtml

18. Yuan S, Carter P, Vithayathil M, et al. Iron Status and Cancer Risk in UK Biobank: A Two-Sample Mendelian Randomization Study. *Nutrients* 2020;12(2) doi: 10.3390/nu12020526 [published Online First: 2020/02/26]
19. Zaitsu M, Kaneko R, Takeuchi T, et al. Occupational class and male cancer incidence: Nationwide, multicenter, hospital-based case-control study in Japan. *Cancer Med* 2019;8(2):795-813. doi: 10.1002/cam4.1945 [published Online First: 2019/01/05]
20. Brenner H, Rothenbacher D, Arndt V. Epidemiology of stomach cancer. *Methods Mol Biol* 2009;472:467-77. doi: 10.1007/978-1-60327-492-0_23 [published Online First: 2008/12/25]
21. Bagnardi V, Rota M, Botteri E, et al. Alcohol consumption and site-specific cancer risk: a comprehensive dose-response meta-analysis. *Br J Cancer* 2015;112(3):580-93. doi: 10.1038/bjc.2014.579 [published Online First: 2014/11/26]
22. Yuan S, Kar S, Vithayathil M, et al. Causal associations of thyroid function and dysfunction with overall, breast and thyroid cancer: A two-sample Mendelian randomization study. *Int J Cancer* 2020 doi: 10.1002/ijc.32988 [published Online First: 2020/03/28]
23. Singletary SE. Rating the risk factors for breast cancer. *Ann Surg* 2003;237(4):474-82. doi: 10.1097/01.sla.0000059969.64262.87 [published Online First: 2003/04/05]
24. Sadeghi H, Rafei M, Bahrami M, et al. Attributable risk fraction of four lifestyle risk factors of thyroid cancer: a meta-analysis. *J Public Health (Oxf)* 2018;40(2):e91-e98. doi: 10.1093/pubmed/fdx088 [published Online First: 2017/10/05]
25. Dal Maso L, Bosetti C, La Vecchia C, et al. Risk factors for thyroid cancer: an epidemiological review focused on nutritional factors. *Cancer Causes Control* 2009;20(1):75-86. doi: 10.1007/s10552-008-9219-5 [published Online First: 2008/09/04]
26. Xie SH, Chen J, Zhang B, et al. Time trends and age-period-cohort analyses on incidence rates of thyroid cancer in Shanghai and Hong Kong. *BMC Cancer* 2014;14:975. doi: 10.1186/1471-2407-14-975 [published Online First: 2014/12/19]
27. Wen D, Zou W, Wen X, et al. Urban-rural disparity in colorectal cancer incidence and increasing trend in relation to socioeconomic development and urbanization in China. *J Int Med Res* 2018;46(10):4181-96. doi: 10.1177/0300060518791090 [published Online First: 2018/08/21]
28. Zhang SK, Zheng R, Chen Q, et al. Oral cancer incidence and mortality in China, 2011. *Chin J Cancer Res* 2015;27(1):44-51. doi: 10.3978/j.issn.1000-9604.2015.01.03 [published Online First: 2015/02/27]
29. Cornish AJ, Law PJ, Timofeeva M, et al. Modifiable pathways for colorectal cancer: a mendelian randomisation analysis. *Lancet Gastroenterol Hepatol* 2020;5(1):55-62. doi: 10.1016/s2468-1253(19)30294-8 [published Online First: 2019/11/02]
30. Ram H, Sarkar J, Kumar H, et al. Oral cancer: risk factors and molecular pathogenesis. *J Maxillofac Oral Surg* 2011;10(2):132-7. doi: 10.1007/s12663-011-0195-z [published Online First: 2012/06/02]
31. Scelo G, Larose TL. Epidemiology and Risk Factors for Kidney Cancer. *J Clin Oncol* 2018:Jco2018791905. doi: 10.1200/jco.2018.79.1905 [published Online First: 2018/10/30]

For peer review only - http://bmjopen.bmj.com/site/about/guidelines.xhtml

Figure legends

Figure 1. Rate differences and 95% confidence intervals in cancer incidence comparing women (A) and men (B) in rural versus urban areas in China, 2008-2012

Figure 2. Rate ratios and 95% confidence intervals in cancer incidence comparing women (A) and men (B) in rural versus urban areas in China, 2008-2012

For peer review only

Table 1. Cancer incidence rates by anatomic site in women in China, 2008-2012

Cancer type	Rural areas			Urban areas		
	Number of cases	Crude Rate (95% CI)	Age-standardized rate (95% CI)	Number of cases	Crude Rate (95% CI)	Age-standardized rate (95% CI)
Lip, oral cavity and pharynx	850	3.7 (3.4, 3.9)	2.8 (2.6, 3.0)	8,329	6.2 (6.1, 6.3)	4.3 (4.2, 4.4)
Esophagus	8,408	36.2 (35.4, 36.9)	26.3 (25.8, 26.9)	6,034	4.5 (4.4, 4.6)	2.7 (2.6, 2.8)
Stomach	8,410	36.2 (35.4, 36.9)	26.5 (25.9, 27.1)	22,267	16.6 (16.4, 16.8)	10.5 (10.4, 10.7)
Small intestine	162	0.7 (0.6, 0.8)	0.5 (0.4, 0.6)	1,818	1.4 (1.3, 1.4)	0.9 (0.8, 0.9)
Colon, rectum and anus	4,268	18.4 (17.8, 18.9)	13.5 (13.1, 13.9)	47,907	35.7 (35.4, 36.1)	22.3 (22.1, 22.5)
Liver and biliary passages	6,084	26.2 (25.5, 26.8)	19.2 (18.8, 19.7)	24,268	18.1 (17.9, 18.3)	11.2 (11.1, 11.3)
Pancreas	1,555	6.7 (6.4, 7.0)	4.8 (4.6, 5.0)	10,067	7.5 (7.4, 7.7)	4.6 (4.5, 4.6)
Larynx	81	0.3 (0.3, 0.4)	0.3 (0.2, 0.3)	508	0.4 (0.3, 0.4)	0.2 (0.2, 0.3)
Lung	7,208	31.0 (30.3, 31.7)	22.6 (22.0, 23.1)	54,148	40.4 (40.1, 40.7)	25.0 (24.8, 25.2)
Bone	448	1.9 (1.8, 2.1)	1.6 (1.4, 1.7)	1,665	1.2 (1.2, 1.3)	1.0 (0.9, 1.0)
Melanoma of skin	109	0.5 (0.4, 0.6)	0.4 (0.3, 0.4)	905	0.7 (0.6, 0.7)	0.4 (0.4, 0.5)
Skin, excluding melanoma	563	2.4 (2.2, 2.6)	1.7 (1.6, 1.9)	5,353	4.0 (3.9, 4.1)	2.4 (2.4, 2.5)
Mesothelioma	31	0.1 (0.1, 0.2)	0.1 (0.1, 0.1)	319	0.2 (0.2, 0.3)	0.2 (0.1, 0.2)
Breast	6,539	28.1 (27.4, 28.8)	21.5 (21.0, 22.0)	80,535	60.1 (59.7, 60.5)	41.3 (41, 41.6)
Uterus	5,572	24 (23.3, 24.6)	18.4 (17.9, 18.8)	34,763	25.9 (25.7, 26.2)	18.1 (17.9, 18.3)
Ovary	1,269	5.5 (5.2, 5.8)	4.2 (4.0, 4.5)	12,782	9.5 (9.4, 9.7)	6.7 (6.6, 6.8)
Other female genital organs	200	0.9 (0.7, 1.0)	0.7 (0.6, 0.7)	2,038	1.5 (1.5, 1.6)	1.0 (1.0, 1.1)
Kidney	380	1.6 (1.5, 1.8)	1.3 (1.2, 1.4)	5,642	4.2 (4.1, 4.3)	2.8 (2.8, 2.9)
Bladder	507	2.2 (2.0, 2.4)	1.6 (1.5, 1.7)	5,029	3.8 (3.6, 3.9)	2.3 (2.2, 2.3)
Other urinary organs	73	0.3 (0.2, 0.4)	0.2 (0.2, 0.3)	2,271	1.7 (1.6, 1.8)	1.0 (1.0, 1.1)
Eye	51	0.2 (0.2, 0.3)	0.2 (0.1, 0.3)	217	0.2 (0.1, 0.2)	0.2 (0.2, 0.2)

For peer review only - http://bmjopen.bmj.com/site/about/guidelines.xhtml

Brain and nervous system	1,405	6.0 (5.7, 6.4)	4.9 (4.6, 5.1)	7,457	5.6 (5.4, 5.7)	4.2 (4.1, 4.3)
Thyroid	999	4.3 (4.0, 4.6)	3.4 (3.2, 3.7)	22,869	17.1 (16.8, 17.3)	13.0 (12.8, 13.2)
Other endocrine organs	165	0.7 (0.6, 0.8)	0.6 (0.5, 0.7)	1,229	0.9 (0.9, 1.0)	0.7 (0.7, 0.8)
Hodgkin lymphoma	42	0.2 (0.1, 0.2)	0.2 (0.1, 0.2)	508	0.4 (0.3, 0.4)	0.3 (0.3, 0.4)
Non-Hodgkin lymphoma	862	3.7 (3.5, 4.0)	2.9 (2.7, 3.1)	7,672	5.7 (5.6, 5.9)	3.9 (3.8, 4.0)
Multiple myeloma	185	0.8 (0.7, 0.9)	0.6 (0.5, 0.7)	2,162	1.6 (1.5, 1.7)	1.0 (1.0, 1.1)
Lymphoid leukemia	1,189	5.1 (4.8, 5.4)	4.6 (4.3, 4.8)	6,565	4.9 (4.8, 5.0)	4.2 (4.1, 4.3)
Other and unspecified	961	4.1 (3.9, 4.4)	3.2 (3.0, 3.4)	13,590	10.1 (10.0, 10.3)	6.7 (6.6, 6.8)
All sites but skin	58,013	249.5 (247.5, 251.5)	186.9 (185.4, 188.4)	383,564	286.2 (285.3, 287.1)	190.9 (190.3, 191.5)
All sites	58,576	251.9 (249.9, 253.9)	188.6 (187.1, 190.2)	388,917	290.2 (289.3, 291.1)	193.4 (192.7, 194)

CI, confidence interval.

For peer review only

Table 2. Cancer incidence rate by anatomic site in men in China, 2008-2012

Cancer type	Rural areas			Urban areas		
	Number of cases	Crude Rate (95% CI)	Age-standardized rate (95% CI)	Number of cases	Crude Rate (95% CI)	Age-standardized rate (95% CI)
Lip, oral cavity and pharynx	1,517	6.3 (6.0, 6.6)	5.0 (4.8, 5.3)	18,962	14.2 (14.0, 14.4)	10.1 (10.0, 10.2)
Esophagus	13,139	54.5 (53.6, 55.4)	43.5 (42.7, 44.2)	21,961	16.5 (16.2, 16.7)	11.1 (11.0, 11.3)
Stomach	18,515	76.8 (75.7, 77.9)	61.3 (60.4, 62.2)	45,179	33.9 (33.5, 34.2)	23.0 (22.8, 23.2)
Small intestine	190	0.8 (0.7, 0.9)	0.6 (0.5, 0.7)	2,238	1.7 (1.6, 1.7)	1.2 (1.1, 1.2)
Colon, rectum and anus	5,121	21.2 (20.7, 21.8)	17.1 (16.6, 17.6)	60,205	45.1 (44.8, 45.5)	30.7 (30.4, 30.9)
Liver and biliary passages	13,885	57.6 (56.7, 58.6)	45.8 (45.1, 46.6)	56,100	42.0 (41.7, 42.4)	29.0 (28.7, 29.2)
Pancreas	1,802	7.5 (7.1, 7.8)	6.0 (5.8, 6.3)	12,593	9.4 (9.3, 9.6)	6.4 (6.3, 6.5)
Larynx	522	2.2 (2.0, 2.4)	1.7 (1.6, 1.9)	5,679	4.3 (4.1, 4.4)	2.9 (2.8, 3.0)
Lung	15,740	65.3 (64.3, 66.3)	52.7 (51.9, 53.5)	104,382	78.2 (77.7, 78.7)	52.8 (52.5, 53.1)
Bone	612	2.5 (2.3, 2.7)	2.2 (2.0, 2.4)	2,041	1.5 (1.5, 1.6)	1.2 (1.2, 1.3)
Melanoma of skin	119	0.5 (0.4, 0.6)	0.4 (0.3, 0.5)	922	0.7 (0.6, 0.7)	0.5 (0.5, 0.5)
Skin, excluding melanoma	542	2.2 (2.1, 2.4)	1.9 (1.7, 2.0)	5,611	4.2 (4.1, 4.3)	2.9 (2.8, 3.0)
Mesothelioma	30	0.1 (0.1, 0.2)	0.1 (0.1, 0.1)	477	0.4 (0.3, 0.4)	0.2 (0.2, 0.3)
Breast	68	0.3 (0.2, 0.4)	0.2 (0.2, 0.3)	677	0.5 (0.5, 0.5)	0.4 (0.3, 0.4)
Prostate	1,152	4.8 (4.5, 5.1)	4.0 (3.8, 4.3)	25,963	19.5 (19.2, 19.7)	13.0 (12.8, 13.2)
Testis	103	0.4 (0.3, 0.5)	0.4 (0.3, 0.5)	1,176	0.9 (0.8, 0.9)	0.8 (0.8, 0.9)
Other male genital organs	184	0.8 (0.7, 0.9)	0.6 (0.5, 0.7)	1,371	1.0 (1.0, 1.1)	0.7 (0.7, 0.8)
Kidney	535	2.2 (2.0, 2.4)	1.8 (1.7, 2.0)	11,201	8.4 (8.2, 8.6)	5.9 (5.8, 6.0)
Bladder	1,610	6.7 (6.4, 7.0)	5.5 (5.2, 5.7)	16,013	12.0 (11.8, 12.2)	8.1 (8.0, 8.2)
Other urinary organs	121	0.5 (0.4, 0.6)	0.4 (0.3, 0.5)	2,449	1.8 (1.8, 1.9)	1.2 (1.2, 1.3)
Eye	49	0.2 (0.2, 0.3)	0.2 (0.1, 0.3)	236	0.2 (0.2, 0.2)	0.2 (0.2, 0.2)
Brain and nervous system	1,496	6.2 (5.9, 6.5)	5.3 (5.0, 5.5)	7,454	5.6 (5.5, 5.7)	4.4 (4.3, 4.5)

For peer review only - http://bmjopen.bmj.com/site/about/guidelines.xhtml

For peer review only

Thyroid	293	1.2 (1.1, 1.4)	1.0 (0.9, 1.1)	7,193	5.4 (5.3, 5.5)	4.2 (4.1, 4.3)
Other endocrine organs	130	0.5 (0.5, 0.6)	0.5 (0.4, 0.5)	1,221	0.9 (0.9, 1.0)	0.8 (0.7, 0.8)
Hodgkin lymphoma	63	0.3 (0.2, 0.3)	0.2 (0.2, 0.3)	740	0.6 (0.5, 0.6)	0.5 (0.4, 0.5)
Non-Hodgkin lymphoma	1,266	5.3 (5.0, 5.5)	4.3 (4.1, 4.6)	10,025	7.5 (7.4, 7.7)	5.5 (5.4, 5.6)
Multiple myeloma	286	1.2 (1.1, 1.3)	0.9 (0.8, 1.1)	3,096	2.3 (2.2, 2.4)	1.6 (1.5, 1.6)
Lymphoid leukemia	1,433	5.9 (5.6, 6.3)	5.4 (5.1, 5.7)	8,751	6.6 (6.4, 6.7)	5.8 (5.6, 5.9)
Other and unspecified	1,186	4.9 (4.6, 5.2)	4.1 (3.9, 4.3)	16,018	12.0 (11.8, 12.2)	8.5 (8.4, 8.7)
All sites but skin	81,167	336.7 (334.4, 339.1)	271.5 (269.6, 273.4)	444,323	333 (332, 333.9)	230.5 (229.8, 231.2)
All sites	81,709	339.0 (336.7, 341.3)	273.4 (271.5, 275.2)	449,934	337.2 (336.2, 338.1)	233.4 (232.7, 234.1)

CI, confidence interval.

For peer review only

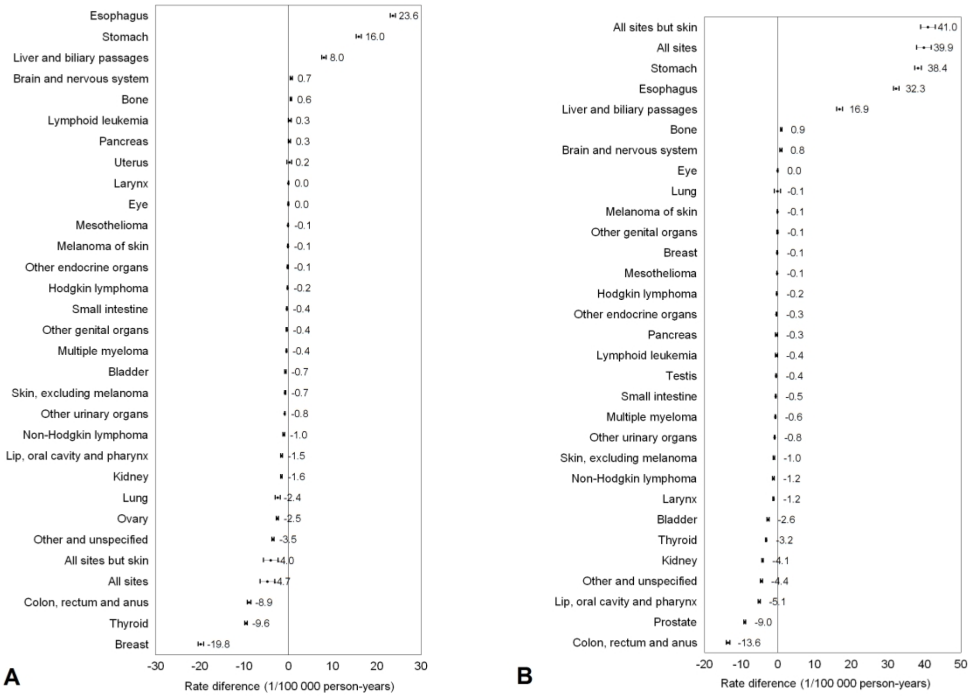

Figure 1. Rate differences and 95% confidence intervals in cancer incidence comparing women (A) and men (B) in rural versus urban areas in China, 2008-2012

721x524mm (96 x 96 DPI)

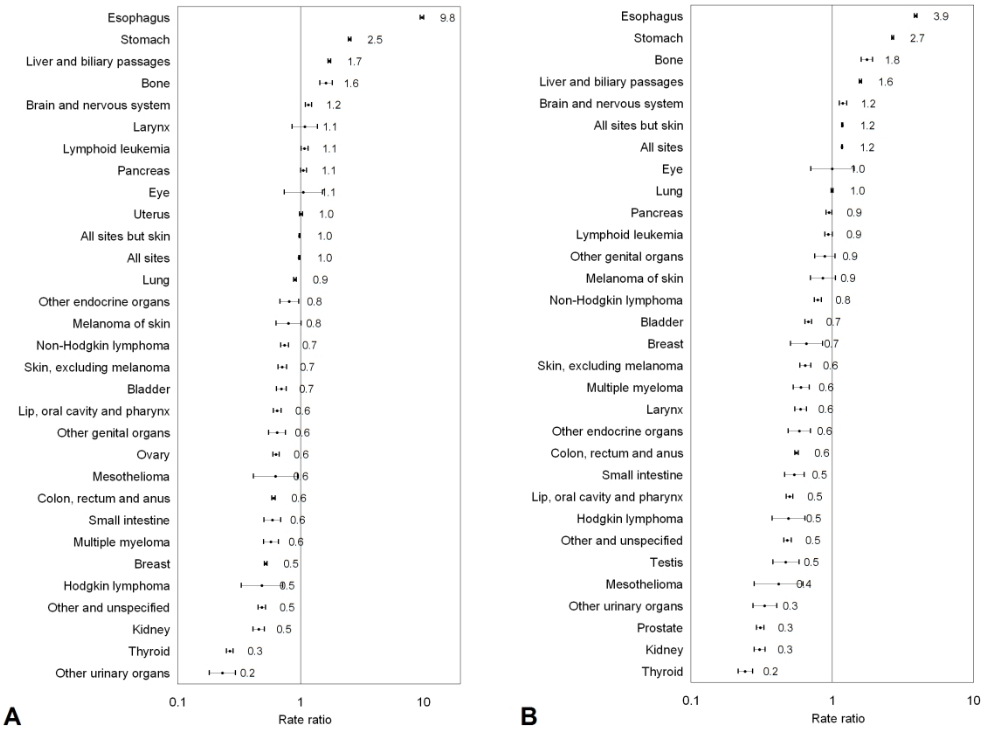

Figure 2. Rate ratios and 95% confidence intervals in cancer incidence comparing women (A) and men (B) in rural versus urban areas in China, 2008-2012

677x514mm (96 x 96 DPI)

Supporting information for

Urban-rural disparity in cancer incidence in China, 2008-2012: A cross-sectional analysis of data from 36 cancer registers

Shuai Yuan, Shao-Hua Xie

Supplementary Table 1. Chinese cancer registries in the Cancer Incidence in Five Continents, XI volume

Supplementary Table 2. Data quality of Chinese cancer registries in the Cancer Incidence in Five Continents, XI volume

For peer review only

Supplementary Table 1. Chinese cancer registries in the Cancer Incidence in Five Continents, XI volume

Region	Registry	Calendar period
Rural areas		
North	Cixian County	2008-2012
North	Shexian County	2008-2012
East	Qidong County	2008-2012
East	Jiashan County	2008-2012
East	Haimen County	2008-2012
East	Jianhu County	2010-2012
East	Guanyun	2008-2012
East	Sheyang	2008-2012
East	Xianju	2010-2012
Central	Linzhou County	2008-2012
Central	Hengdong	2009-2012
Central	Xiping	2010-2012
Central	Yanshi	2010-2012
Southwest	Yanting County	2008-2012
Urban areas		
North	Beijing	2008-2012
Northeast	Anshan City	2008-2012
Northeast	Harbin City, Nangang District	2008-2012
Northeast	Benxi	2008-2011
Northeast	Shenyang	2008-2012
East	Shanghai City	2008-2012
East	Hangzhou City	2008-2012
East	Jiaxing City	2008-2012
East	Hefei	2010-2012
East	Huaiyin District, Huai'an	2009-2012
East	Lianyungang	2008-2012
East	Maanshan	2008-2012
East	Tongling City	2008-2012
East	Wuxi	2010-2012
Central	Wuhan City	2008-2012
Central	Yueyanglou	2009-2012
South	Zhongshan City	2010-2012
South	Guangzhou	2010-2012
South	Hong Kong	2008-2012
South	Jiangmen	2010-2012
South	Liuzhou	2009-2012
South	Zhuhai	2010-2012

For peer review only

Supplementary Table 2. Data quality of Chinese cancer registries in the Cancer Incidence in Five Continents, XI volume

Region		Male				Female			
		Cases	MV (%)	DCO (%)	ASR (W)	Cases	MV (%)	DCO (%)	ASR (W)
**Rural areas**									
North	Cixian County	5,160	79.7	2.5	443.2	3,765	78.5	1.7	280.7
North	Shexian County	3,839	74.0	1.9	343.5	2,297	77.2	2.3	201.9
East	Qidong County	11,544	44.2	0.0	218.4	7,661	58.2	0.0	132.4
East	Jiashan County	4,068	64.5	0.1	224.1	2,999	73.2	0.0	169.4
East	Haimen County	10,521	48.4	0.5	209.6	7,623	62.8	0.5	154.6
East	Jianhu County	4,241	76.9	0.1	253.0	2,792	82.5	0.3	162.4
East	Guanyun	5,924	51.7	1.8	181.6	4,119	65.6	2.1	137.3
East	Sheyang	8,647	63.2	0.3	225.0	6,415	70.7	0.3	168.0
East	Xianju	2,983	64.8	4.5	277.4	1,780	69.8	4.2	174.7
Central	Linzhou County	8,721	78.4	1.5	309.8	7,111	78.2	1.3	220.5
Central	Hengdong	3,266	60.4	7.2	171.8	2,382	71.2	5.0	131.7
Central	Xiping	3,050	63.3	2.5	200.5	2,550	70.3	1.8	160.9
Central	Yanshi	2,167	67.9	3.9	215.2	1,969	69.4	4.2	170.7
Southwest	Yanting County	7,053	77.9	1.4	435.6	4,560	79.5	2.1	256.6
**Urban areas**									
North	Beijing	61,058	71.4	1.9	166.7	58,686	79.1	1.4	165.6
Northeast	Anshan City	12,562	62.4	11.8	210.5	11,581	72.6	10.5	178.8
Northeast	Harbin City, Nangang District	6,509	70.5	3.9	191.9	6,009	78.7	2.5	154.9
Northeast	Benxi	5,171	52.2	6.1	244.0	4,294	64.3	4.3	181.4
Northeast	Shenyang	26,978	61.1	6.8	181.5	25,643	69.9	5.0	157.4
East	Shanghai City	68,195	69.8	0.0	200.9	62,499	75.9	0.0	187.6
East	Hangzhou City	55,665	66.1	0.7	206.2	46,116	73.4	0.5	176.3
East	Jiaxing City	4,605	64.7	0.1	207.1	3,923	75.6	0.2	177.8
East	Hefei	10,092	52.5	8.1	252.7	6,268	58.8	6.2	155.7
East	Huaiyin District, Huai'an	5,846	62.2	8.3	220.7	3,406	70.0	7.8	130.3
East	Lianyungang	5,776	67.9	1.5	185.9	4,422	74.9	1.2	138.2
East	Maanshan	5,417	69.0	0.4	268.8	3,567	76.4	0.5	173.1
East	Tongling City	4,076	75.3	5.0	322.5	2,315	78.0	4.0	174.9
East	Wuxi	13,086	63.0	1.9	205.1	9,544	71.0	1.6	149.2
Central	Wuhan City	36,737	71.0	0.7	205.1	30,701	79.7	0.6	167.6
Central	Yueyanglou	2,396	76.1	2.9	281.0	1,795	82.5	2.8	190.8
South	Zhongshan City	6,828	70.9	0.0	251.9	4,776	83.0	0.0	158.4
South	Guangzhou	33,619	70.0	0.8	204.1	27,801	82.0	0.4	162.2
South	Hong Kong	66,837	84.0	0.7	229.8	60,084	89.7	0.6	189.9
South	Jiangmen	2,890	69.1	0.6	236.3	2,249	83.0	0.5	170.1
South	Liuzhou	5,825	62.3	2.7	224.2	4,437	75.2	2.1	165.5
South	Zhuhai	4,155	62.3	3.7	243.8	3,450	72.2	2.4	194.1

ASR, age-standardized rate; DCO, death certificate only; MV, microscopically verified.

For peer review only

STROBE 2007 (v4) Statement—Checklist of items that should be included in reports of *cross-sectional studies*

Section/Topic	Item #	Recommendation	Reported on page #
**Title and abstract**	1	(*a*) Indicate the study's design with a commonly used term in the title or the abstract	1
		(*b*) Provide in the abstract an informative and balanced summary of what was done and what was found	2
**Introduction**			
Background/rationale	2	Explain the scientific background and rationale for the investigation being reported	4
Objectives	3	State specific objectives, including any prespecified hypotheses	4
**Methods**			
Study design	4	Present key elements of study design early in the paper	5
Setting	5	Describe the setting, locations, and relevant dates, including periods of recruitment, exposure, follow-up, and data collection	5
Participants	6	(*a*) Give the eligibility criteria, and the sources and methods of selection of participants	5
Variables	7	Clearly define all outcomes, exposures, predictors, potential confounders, and effect modifiers. Give diagnostic criteria, if applicable	5
Data sources/ measurement	8*	For each variable of interest, give sources of data and details of methods of assessment (measurement). Describe comparability of assessment methods if there is more than one group	5
Bias	9	Describe any efforts to address potential sources of bias	*5*
Study size	10	Explain how the study size was arrived at	5
Quantitative variables	11	Explain how quantitative variables were handled in the analyses. If applicable, describe which groupings were chosen and why	5
Statistical methods	12	(*a*) Describe all statistical methods, including those used to control for confounding	5
		(*b*) Describe any methods used to examine subgroups and interactions	n/a
		(*c*) Explain how missing data were addressed	n/a
		(*d*) If applicable, describe analytical methods taking account of sampling strategy	n/a
		(*e*) Describe any sensitivity analyses	n/a
**Results**			

For peer review only

Participants	13*	(a) Report numbers of individuals at each stage of study—eg numbers potentially eligible, examined for eligibility, confirmed eligible, included in the study, completing follow-up, and analysed	6
		(b) Give reasons for non-participation at each stage	n/a
		(c) Consider use of a flow diagram	n/a
Descriptive data	14*	(a) Give characteristics of study participants (eg demographic, clinical, social) and information on exposures and potential confounders	6-8
		(b) Indicate number of participants with missing data for each variable of interest	n/a
Outcome data	15*	Report numbers of outcome events or summary measures	6-8
Main results	16	(*a*) Give unadjusted estimates and, if applicable, confounder-adjusted estimates and their precision (eg, 95% confidence interval). Make clear which confounders were adjusted for and why they were included	6-8
		(*b*) Report category boundaries when continuous variables were categorized	
		(*c*) If relevant, consider translating estimates of relative risk into absolute risk for a meaningful time period	n/a
Other analyses	17	Report other analyses done—eg analyses of subgroups and interactions, and sensitivity analyses	n/a
**Discussion**			
Key results	18	Summarise key results with reference to study objectives	8
Limitations	19	Discuss limitations of the study, taking into account sources of potential bias or imprecision. Discuss both direction and magnitude of any potential bias	8-9
Interpretation	20	Give a cautious overall interpretation of results considering objectives, limitations, multiplicity of analyses, results from similar studies, and other relevant evidence	8-9
Generalisability	21	Discuss the generalisability (external validity) of the study results	8-9
**Other information**			
Funding	22	Give the source of funding and the role of the funders for the present study and, if applicable, for the original study on which the present article is based	12

*Give information separately for cases and controls in case-control studies and, if applicable, for exposed and unexposed groups in cohort and cross-sectional studies.

Note: An Explanation and Elaboration article discusses each checklist item and gives methodological background and published examples of transparent reporting. The STROBE checklist is best used in conjunction with this article (freely available on the Web sites of PLoS Medicine at http://www.plosmedicine.org/, Annals of Internal Medicine at http://www.annals.org/, and Epidemiology at http://www.epidem.com/). Information on the STROBE Initiative is available at www.strobe-statement.org.

For peer review only

BMJ Open

Urban-rural disparity in cancer incidence in China, 2008-2012: A cross-sectional analysis of data from 36 cancer registers

Journal:	*BMJ Open*
Manuscript ID	bmjopen-2020-042762.R2
Article Type:	Original research
Date Submitted by the Author:	14-Apr-2021
Complete List of Authors:	Yuan, Shuai; Karolinska Institutet, Institute of Environmental Medicine<br>Xie, Shao-Hua; Karolinska University Hospital, Department of Molecular Medicine and Surgery
**Primary Subject Heading**:	Epidemiology
Secondary Subject Heading:	Oncology, Public health
Keywords:	EPIDEMIOLOGY, ONCOLOGY, PUBLIC HEALTH

SCHOLARONE™
Manuscripts

I, the Submitting Author has the right to grant and does grant on behalf of all authors of the Work (as defined in the below author licence), an exclusive licence and/or a non-exclusive licence for contributions from authors who are: i) UK Crown employees; ii) where BMJ has agreed a CC-BY licence shall apply, and/or iii) in accordance with the terms applicable for US Federal Government officers or employees acting as part of their official duties; on a worldwide, perpetual, irrevocable, royalty-free basis to BMJ Publishing Group Ltd ("BMJ") its licensees and where the relevant Journal is co-owned by BMJ to the co-owners of the Journal, to publish the Work in this journal and any other BMJ products and to exploit all rights, as set out in our licence.

The Submitting Author accepts and understands that any supply made under these terms is made by BMJ to the Submitting Author unless you are acting as an employee on behalf of your employer or a postgraduate student of an affiliated institution which is paying any applicable article publishing charge ("APC") for Open Access articles. Where the Submitting Author wishes to make the Work available on an Open Access basis (and intends to pay the relevant APC), the terms of reuse of such Open Access shall be governed by a Creative Commons licence – details of these licences and which Creative Commons licence will apply to this Work are set out in our licence referred to above.

Other than as permitted in any relevant BMJ Author's Self Archiving Policies, I confirm this Work has not been accepted for publication elsewhere, is not being considered for publication elsewhere and does not duplicate material already published. I confirm all authors consent to publication of this Work and authorise the granting of this licence.

For peer review only

Urban-rural disparity in cancer incidence in China, 2008-2012: A cross-sectional analysis of data from 36 cancer registers

Running head: Cancer disparity in China

Shuai Yuan, B.Med, MMedSc[1], Shao-Hua Xie, MD, PhD[2*]

Affiliations

[1] Unit of Cardiovascular and Nutritional Epidemiology, Institute of Environmental Medicine, Karolinska Institutet, Stockholm, Sweden.

[2] Upper Gastrointestinal Surgery, Department of Molecular Medicine and Surgery, Karolinska Institutet, Karolinska University Hospital, Stockholm, Sweden

[*]Correspondence

Shao-Hua Xie, MD, PhD, Upper Gastrointestinal Surgery, Department of Molecular medicine and Surgery, Karolinska Institutet, Karolinska University Hospital, Retzius väg 13a, 4th Floor, 17 177 Stockholm, Sweden. Email: shaohua.xie@ki.se

Words of Abstract / Manuscript: 224 / 2286

Number of tables / figures: 2 / 2

For peer review only

Abstract

Objective: The substantial differences in socioeconomic and lifestyle exposures between urban and rural areas in China may lead to urban-rural disparity in cancer risk. This study aimed to assess the urban-rural disparity in cancer incidence in China.

Methods: Using data from 36 regional cancer registries in China in 2008-2012, we compared the age-standardized incidence rates of cancer by sex and anatomic site between rural and urban areas. We calculated the rate difference and rate ratio comparing rates in rural versus urban areas by sex and cancer type.

Results The incidence rate of all cancers in women was slightly lower in rural areas than in urban areas, but the total cancer rate in men was higher in rural areas than in urban areas. The incidence rates in women were higher in rural areas than in urban areas for cancers of the esophagus, stomach, and liver and biliary passages, but lower for cancers of thyroid and breast. Men residing in rural areas had higher incidence rates for cancers of the esophagus, stomach, and liver and biliary passages, but lower rates for prostate cancer, lip, oral cavity and pharynx cancer, and colorectal cancer.

Conclusions: Our findings suggest substantial urban-rural disparity in cancer incidence in China, which varies across cancer types and the sexes. Cancer prevention strategies should be tailored for common cancers in rural and urban areas.

Keywords: Neoplasms; incidence; socioeconomic status; spatial distribution; epidemiology

For peer review only - http://bmjopen.bmj.com/site/about/guidelines.xhtml

Strengths and limitations of this study

- We used data from the 36 cancer registers of high quality to investigate urban-rural disparity for overall and site-specific cancer incidence by sex and on both absolute and relative scales.
- The cross-sectional design limited the causal inference in geographical disparities to cancer incidence.
- Included registries only represented a limited proportion of the total population in China and were not in well balanced distribution regarding geographic regions.
- We were not able to analyze the data by histological type.
- We could not evaluate the associations of cancer disparity with gross domestic product, human development index or corresponding risk factors.

For peer review only

Introduction

Cancer is one of the leading causes of death worldwide, including in China. According to China Health Statistics Yearbook, an estimated 2.2 million cancer deaths occurred in China in 2017, accounting for around 25% of all deaths in this year [1]. Moreover, the past few decades have witnessed rapid urbanization and industrialization in China, and the uneven distribution of wealth and lifestyle profiles across regions in the nation during this process may have in turn resulted in substantial urban-rural disparity in cancer risk. Previous studies have suggested marked urban-rural disparity in cancer incidence in China, and such disparity seems varying across cancer types [2-8]. However, most of these previous studies were limited in a certain area or province, or specific types of cancer only. Due to historically limited population-based cancer registry data in China, the urban-rural disparity in cancer incidence has not been well characterized.

China has recently established the National Central Cancer Registry and data from 36 regional cancer registries have been included in the latest release of *Cancer Incidence in Five Continents* series published by the International Agency for Research on Cancer [9]. This provides a new opportunity to assess the regional disparity in cancer incidence in China on a national scale. Therefore, using data from these high-quality registries, we conducted the present study to characterize the urban-rural disparity in total and type-specific cancer incidence by sex in China in 2008-2012.

Methods

Data source

We extracted data on cancer incidence and population sizes from 36 regional cancer registries, including 14 registries in rural areas and 22 in urban areas, in China during 2008-2012 which are

For peer review only - http://bmjopen.bmj.com/site/about/guidelines.xhtml

included in the XI volume of *Cancer Incidence in Five Continents* series published by the International Agency for Research on Cancer [10]. These registries are located in different geographical regions throughout the country. Cancer cases were defined by International Classification of Disease codes, which are listed in a webpage of the International Agency for Research on Cancer (https://ci5.iarc.fr/CI5-XI/PDF/Chapter%203.pdf). Detailed information on the included registries is presented in **Supplementary Table 1 and Table 2**. The case-weighted means of percentage of case defined by death certificate only were ~1.9 in male, ~1.5 in female and ~1.7 in both male and female among included registries. Included population from 36 cancer registries made up ~5% of the total population in China. We pooled the numbers of cases and population sizes at risk from multiple registers separately for urban and rural areas by sex and cancer type (anatomic site).

Statistical analysis

We calculated the crude and age-standardized incidence rates (ASRs) and their 95% confidence intervals (CIs) by sex and cancer type separately for rural and urban areas. The ASRs were computed by using the direct standardization method with World Standard Population 2000 as reference [11]. The 95% CIs of crude rates were estimated under the assumption of Poisson distribution and CIs for ASRs were calculated based on the gamma distribution assuming that the standardized rate is a weighted sum of independent Poisson random variables [12]. The urban-rural disparities in cancer incidence were quantitatively assessed with two disparity measures (i.e., rate difference (RD) on the absolute scale and rate ratio (RR) on the relative scale) [13]. We calculated the RD (ASR in rural areas – ASR in urban areas) and rate ratio (ratio of ASRs in rural areas

For peer review only - http://bmjopen.bmj.com/site/about/guidelines.xhtml

relative to urban areas) with 95% CIs by sex and cancer type. All statistical analyses were performed using the SAS version 9.4 (SAS Institute, Cary, NC) and two-sided.

Patient and Public Involvement

Patients or the public were not involved in the design, or conduct, or reporting, or dissemination plans of our research.

Ethical considerations

The analyses were solely based on publicly available data of population sizes and aggregated number of cancer cases and as such, ethics approval or consent to participate was not deemed to be necessary. No individual-level data were involved in the study or in defining the research question or outcome measures.

Results

Cancer incidence in rural areas

The crude rates and ASRs in rural and urban areas are displayed in **Table 1** for women and in **Table 2** for men. In total, 58,576 female and 81,709 male new cancer cases were recorded in 14 cancer registers in rural areas in China during the period 2008-2012, contributing to the ASRs of 188.6 (95% CI, 187.1, 190.2) per 100,000 population per year in women and 273.4 (95% CI, 271.5, 275.2) per 100,000 population per year in men. In women residing in rural areas, the ASRs were observed for stomach cancer, followed by cancers of the esophagus, lung, breast, and liver and biliary passages. In men in rural areas, stomach cancer was also the most common cancer type, followed by cancers of the lung, liver and biliary passages, esophagus, and colon, rectum and anus.

For peer review only - http://bmjopen.bmj.com/site/about/guidelines.xhtml

Cancer incidence in urban areas

A total of 388,917 women and 449,934 men were diagnosed with any cancer as recorded in the 22 registers in urban areas in China during the period 2008-2012. The ASRs of all cancers were 193.4 (95% CI, 192.7, 194.0) per 100,000 population per year in women and 233.4 (95% CI, 232.7, 234.1) per 100,000 population per year in men. The five most frequent cancers in women in urban areas were cancers of the breast, lung, colon and rectum, uterus, and thyroid. In men, the highest ASRs were observed for lung cancer, followed by cancer of colon and rectum, liver and biliary passages, stomach, and prostate.

Rate difference comparing rural and urban areas

Figure 1 presents the absolute difference in cancer incidence, i.e., RDs comparing rural and urban areas, in women and in men. The total cancer incidence was lower in women (RD, -4.7 per 100,000 population per year, 95% CI, -6.4, -3.1) but higher in men (RD, 39.9, 95% CI, 39.0, 43.0) living in the rural areas than those in urban areas. Compared with those in urban areas, women living in rural areas had substantially higher incidence rates of cancers of the esophagus, stomach, and liver and biliary passages cancer, but lower incidence rates of cancers of the breast, thyroid, colon and rectum, ovary, and lung. Men residing in the rural areas had higher incidence rates of cancers of the stomach, esophagus, and liver and biliary passages, while those in urban areas had higher rates of cancers of colon and rectum, prostate cancer, and lip, oral cavity and pharynx, kidney, thyroid, and bladder.

Rate ratio comparing rural and urban areas

For peer review only - http://bmjopen.bmj.com/site/about/guidelines.xhtml

The RRs measuring the urban-rural disparity cancer incidence on the relative scale are shown in **Figure 2**. The total cancer incidence rates were similar between women residing in rural areas and those in urban areas as measured by RR (0.98, 95% CI, 0.97, 0.98). However, the total cancer incidence rate in men in rural areas was around 20% higher than those in urban areas (1.17, 95% CI, 1.16, 1.18). When analyzed by cancer type, compared those in urban areas, women in rural areas had substantially higher incidence rates for cancers of the esophagus and stomach, but at least 50% lower incidence rates for cancers of urinary organs other than kidney and bladder, thyroid cancer, kidney cancer, Hodgkin lymphoma, and breast cancer. Men in rural areas had higher incidence rates of cancers of the esophagus and stomach, but 50% or lower incidence rates of cancers of the thyroid, kidney, prostate, mesothelioma, testis, Hodgkin lymphoma, lip, oral cavity and pharynx, and small intestine.

Discussion

The present study revealed substantial disparities in cancer incidence between rural and urban areas in China, and the patterns varied across cancer types and the sexes. The overall cancer incidence was higher in men living in rural areas compared with those in urban areas but showed less urban-rural disparity in women. Both men and women in rural areas had substantially higher incidence rates of cancers of the esophagus, stomach, and liver and biliary passages, while higher incidence rates were observed in urban areas for a number of other cancers, including cancers of colon and rectum, thyroid, breast, prostate, lip, oral cavity and pharynx, and kidney. This study provided an updated assessment of the urban-rural disparity in cancer incidence in China on a nationwide scale.

For peer review only - http://bmjopen.bmj.com/site/about/guidelines.xhtml

Overall, our findings were consistent with previous reports. A study using data from 72 registries in the Chinese National Central Cancer Registry in 2009 showed that esophageal cancer was one of the most common cancers in China, and the incidence rate was particularly high in men in rural areas [3]. A more recent study including 177 cancer registries further confirmed the higher incidence and mortality rates of esophageal cancer in rural areas than in urban areas in both sexes 2011 [4]. In this study, we found that the incidence rate of esophageal cancer in women s was 9.8 times higher in rural arear than in urban areas, and men in rural areas also had 3.9 times higher risk of esophageal cancer than those in urban areas. Such striking urban-rural disparity may be partially explained the higher prevalence of major risk factors for esophageal cancer, including lower socioeconomic status, tobacco smoking, heavy alcohol use, and dietary factors, in rural areas [14 15]. On the other hand, the etiology of esophageal cancer, particularly that of the main histological subtype esophageal squamous cell carcinoma in China, has not been fully elucidated [16]. There may be other mechanisms explaining the burden gap between rural and urban populations in China.

Higher incidence rates of stomach and liver cancers in rural areas than in urban areas in China have also been reported previously [5 6]. Our findings were in line with these reports, which stressed the need for targeted prevention strategies for these cancers in rural areas. As many modifiable factors, including obesity [17], nutrition [18], Helicobacter pylori infection [19], virus infection [20], alcohol use [21] and smoking [20] are important risk factors for stomach and/or liver cancers, primary prevention strategy targeting at these dimensions may be implemented in rural areas to narrow the urban-rural disparity in these cancers.

Thyroid and breast cancers are the most common cancer types in women, especially in the middle-aged group [22]. Higher incidence rates of thyroid and breast cancers in women in urban areas than in rural areas in China have been previously reported [7 8], which were in in line with our

For peer review only - http://bmjopen.bmj.com/site/about/guidelines.xhtml

findings. Such urban-rural disparity might be contributable to the possible differences in prevalence of risk factors, e.g., smoking, obesity, radiation exposure, oral contraceptives usage and intake of vegetables [23-25], between women in rural and urban areas. Another potential explanation is the more frequent use of screening diagnostic procedures, such as ultrasound, Doppler examination, CT and MRI scanning, and biochemical markers, in urban residents, which might have increased the detection of these cancers at an early stage, and thus, led to somewhat overestimated incidence of these cancers in recent years [26]. The more frequent use of screening or diagnostic procedures, such as prostate-specific antigen testing, in urban residents may also partially explain the higher incidence rate of prostate cancer in men living in urban areas than in rural areas.

We found higher incidence rates of colorectal cancer in both men and women living in urban areas compared with their counterparts in rural areas, which were in agreement with some other studies [5 27 28]. Such disparity might be explained by higher prevalence of certain risk factors, particularly those associated with Western lifestyles including low intake of fruit and vegetables, read meat consumption, and lack of physical activity, in those in urban areas. However, it was also likely due to differences in access to diagnostic and treatment services between urban and rural residents. Nevertheless, considering the huge disease burden and possibility of early detection, both primary and secondary prevention strategies are highly needed for targeting urban populations, not only for minimizing the urban-rural disparity but also for reducing deaths from this cancer. A tool tick, comprehensively including lifestyle, obesity, and cardiometabolic factors may be used for prevention of colorectal cancer [29].

The etiology of some other cancers with notable urban-rural disparity, including oral cancer and kidney cancer, has not been well illustrated. A better management regarding tobacco smoking,

For peer review only - http://bmjopen.bmj.com/site/about/guidelines.xhtml

alcohol use, sexually transmitted disease, dental hygiene, nutrition, and occupational hazards in urban residents might facilitate the reduction of disparity in the incidence of these cancers [30 31].

There are strengths of the present study. One major strength was that we used data from the 36 cancer registers of high quality in terms of completeness of coverage and accuracy, thereby lending validity to the findings. In addition, we comprehensively investigated urban-rural disparity for overall and site-specific cancer incidence by sex and on both absolute and relative scales.

This study also has some limitations. This study was embedded in the cross-sectional design, which might not conclude the causation between geographical disparities to cancer incidence. In addition, we used data constructed at the city-level. Thus, whether our findings could be generalized to the individuals needs verification. The included registries only represented a limited proportion of the total population in China and were not in well balanced distribution regarding geographic regions, and thus, the strengths of the disparity measures may not exactly reflect the extent of nationwide urban-rural disparity in cancer incidence. Due to unavailability of complete data in all cancer registries, we were not able to analyze the data by histological type, or the associations of gross domestic product or human development index with ASRs. Last but not least, the analyses were based on cancer incidence data only without information on risk factors, and we could not direct evaluate how the observed disparity could be explained by the corresponding risk factors.

Conclusions

This updated assessment of the urban-rural disparity in cancer incidence in China revealed substantial urban-rural disparity which varies across cancer types and the sexes. The observed urban-rural disparity may be explained by a combination of differential prevalence of risk factors

For peer review only - http://bmjopen.bmj.com/site/about/guidelines.xhtml

and access to screening and diagnostic services. Cancer prevention strategies should be tailored for common cancers in rural and urban areas.

For peer review only

For peer review only - http://bmjopen.bmj.com/site/about/guidelines.xhtml

Additional information

Acknowledgements: We would like to thank International Agency for Research on Cancer for sharing data.

Contributors: SHX designed the study, analyzed the data and reviewed the article. SY analyzed the data and drafted and reviewed the article. All authors read and approved the final manuscript.

Funding: This study was supported by the Swedish Cancer Society (grant number 190043) and Karolinska Institutet Research Foundation (grant number 2018-01558).

Competing interests: None declared.

Patient consent for publication: Not required.

Data availability statement: All data used are publicly available from the International Agency for Research on Cancer.

Ethical Approval Statement: The analyses were solely based on publicly available data of population sizes and aggregated number of cancer cases and as such, ethics approval or consent to participate was not deemed to be necessary.

For peer review only

References

1. Chen W, Xia C, Zheng R, et al. Disparities by province, age, and sex in site-specific cancer burden attributable to 23 potentially modifiable risk factors in China: a comparative risk assessment. *Lancet Glob Health* 2019;7(2):e257-e69. doi: 10.1016/s2214-109x(18)30488-1 [published Online First: 2019/01/27]
2. Pan R, Zhu M, Yu C, et al. Cancer incidence and mortality: A cohort study in China, 2008-2013. *Int J Cancer* 2017;141(7):1315-23. doi: 10.1002/ijc.30825 [published Online First: 2017/06/09]
3. Chen W, He Y, Zheng R, et al. Esophageal cancer incidence and mortality in China, 2009. *J Thorac Dis* 2013;5(1):19-26. doi: 10.3978/j.issn.2072-1439.2013.01.04 [published Online First: 2013/02/02]
4. Zeng H, Zheng R, Zhang S, et al. Esophageal cancer statistics in China, 2011: Estimates based on 177 cancer registries. *Thorac Cancer* 2016;7(2):232-7. doi: 10.1111/1759-7714.12322 [published Online First: 2016/04/05]
5. Yang L, Zheng R, Wang N, et al. Incidence and mortality of stomach cancer in China, 2014. *Chin J Cancer Res* 2018;30(3):291-98. doi: 10.21147/j.issn.1000-9604.2018.03.01 [published Online First: 2018/07/27]
6. Fei FR, Hu RY, Gong WW, et al. Analysis of Mortality and Survival Rate of Liver Cancer in Zhejiang Province in China: A General Population-Based Study. *Can J Gastroenterol Hepatol* 2019;2019:1074286. doi: 10.1155/2019/1074286 [published Online First: 2019/07/31]
7. Wen D, Wen X, Yang Y, et al. Urban rural disparity in female breast cancer incidence rate in China and the increasing trend in parallel with socioeconomic development and urbanization in a rural setting. *Thorac Cancer* 2018;9(2):262-72. doi: 10.1111/1759-7714.12575 [published Online First: 2017/12/28]
8. Yang L, Zheng R, Wang N, et al. [Analysis of incidence and mortality of thyroid cancer in China, 2010]. *Zhonghua Yu Fang Yi Xue Za Zhi* 2014;48(8):663-8. [published Online First: 2014/11/13]
9. CI5 XI - Home 2020 [Available from: https://ci5.iarc.fr/CI5-XI/Default.aspx.
10. Bray F CM, Mery L, Piñeros M, Znaor A, Zanetti R, Ferlay J. Cancer Incidence in Five Continents, Vol. XI (electronic version): Lyon: International Agency for Research on Cancer.; 2017 [Available from: https://ci5.iarc.fr.
11. O.B. Ahmad CB-P, A.D. Lopez, C.J.L. Murray, R. Lozano, M. Inoue. Age Standardization of Rates: A New WHO Standard. GPE Discussion Paper Series: No.31: World Health Organization, Geneva; 2001 [
12. Fay MP, Feuer EJ. Confidence intervals for directly standardized rates: a method based on the gamma distribution. *Stat Med* 1997;16(7):791-801.
13. Xie SH, Rabbani S, Petrick JL, et al. Racial and Ethnic Disparities in the Incidence of Esophageal Cancer in the United States, 1992-2013. *Am J Epidemiol* 2017;186(12):1341-51. doi: 10.1093/aje/kwx221 [published Online First: 2017/06/24]
14. Zhou X, Su Z, Deng H, et al. A comparative survey on alcohol and tobacco use in urban and rural populations in the Huaihua District of Hunan Province, China. *Alcohol* 2006;39(2):87-96. doi: 10.1016/j.alcohol.2006.07.003 [published Online First: 2006/12/01]
15. He YN, Zhai FY, Ge KY. [Measuring diet quality of chinese 18 - 59 years adult using chinese diet balance index]. *Wei Sheng Yan Jiu* 2005;34(4):442-4. [published Online First: 2005/10/19]
16. Xie SH, Lagergren J. Risk factors for oesophageal cancer. *Best Pract Res Clin Gastroenterol* 2018;36-37:3-8. doi: 10.1016/j.bpg.2018.11.008 [published Online First: 2018/12/16]
17. Larsson SC, Wolk A. Overweight, obesity and risk of liver cancer: a meta-analysis of cohort studies. *Br J Cancer* 2007;97(7):1005-8. doi: 10.1038/sj.bjc.6603932 [published Online First: 2007/08/19]

For peer review only - http://bmjopen.bmj.com/site/about/guidelines.xhtml

18. Yuan S, Carter P, Vithayathil M, et al. Iron Status and Cancer Risk in UK Biobank: A Two-Sample Mendelian Randomization Study. *Nutrients* 2020;12(2) doi: 10.3390/nu12020526 [published Online First: 2020/02/26]
19. Zaitsu M, Kaneko R, Takeuchi T, et al. Occupational class and male cancer incidence: Nationwide, multicenter, hospital-based case-control study in Japan. *Cancer Med* 2019;8(2):795-813. doi: 10.1002/cam4.1945 [published Online First: 2019/01/05]
20. Brenner H, Rothenbacher D, Arndt V. Epidemiology of stomach cancer. *Methods Mol Biol* 2009;472:467-77. doi: 10.1007/978-1-60327-492-0_23 [published Online First: 2008/12/25]
21. Bagnardi V, Rota M, Botteri E, et al. Alcohol consumption and site-specific cancer risk: a comprehensive dose-response meta-analysis. *Br J Cancer* 2015;112(3):580-93. doi: 10.1038/bjc.2014.579 [published Online First: 2014/11/26]
22. Yuan S, Kar S, Vithayathil M, et al. Causal associations of thyroid function and dysfunction with overall, breast and thyroid cancer: A two-sample Mendelian randomization study. *Int J Cancer* 2020 doi: 10.1002/ijc.32988 [published Online First: 2020/03/28]
23. Singletary SE. Rating the risk factors for breast cancer. *Ann Surg* 2003;237(4):474-82. doi: 10.1097/01.sla.0000059969.64262.87 [published Online First: 2003/04/05]
24. Sadeghi H, Rafei M, Bahrami M, et al. Attributable risk fraction of four lifestyle risk factors of thyroid cancer: a meta-analysis. *J Public Health (Oxf)* 2018;40(2):e91-e98. doi: 10.1093/pubmed/fdx088 [published Online First: 2017/10/05]
25. Dal Maso L, Bosetti C, La Vecchia C, et al. Risk factors for thyroid cancer: an epidemiological review focused on nutritional factors. *Cancer Causes Control* 2009;20(1):75-86. doi: 10.1007/s10552-008-9219-5 [published Online First: 2008/09/04]
26. Xie SH, Chen J, Zhang B, et al. Time trends and age-period-cohort analyses on incidence rates of thyroid cancer in Shanghai and Hong Kong. *BMC Cancer* 2014;14:975. doi: 10.1186/1471-2407-14-975 [published Online First: 2014/12/19]
27. Wen D, Zou W, Wen X, et al. Urban-rural disparity in colorectal cancer incidence and increasing trend in relation to socioeconomic development and urbanization in China. *J Int Med Res* 2018;46(10):4181-96. doi: 10.1177/0300060518791090 [published Online First: 2018/08/21]
28. Zhang SK, Zheng R, Chen Q, et al. Oral cancer incidence and mortality in China, 2011. *Chin J Cancer Res* 2015;27(1):44-51. doi: 10.3978/j.issn.1000-9604.2015.01.03 [published Online First: 2015/02/27]
29. Cornish AJ, Law PJ, Timofeeva M, et al. Modifiable pathways for colorectal cancer: a mendelian randomisation analysis. *Lancet Gastroenterol Hepatol* 2020;5(1):55-62. doi: 10.1016/s2468-1253(19)30294-8 [published Online First: 2019/11/02]
30. Ram H, Sarkar J, Kumar H, et al. Oral cancer: risk factors and molecular pathogenesis. *J Maxillofac Oral Surg* 2011;10(2):132-7. doi: 10.1007/s12663-011-0195-z [published Online First: 2012/06/02]
31. Scelo G, Larose TL. Epidemiology and Risk Factors for Kidney Cancer. *J Clin Oncol* 2018:Jco2018791905. doi: 10.1200/jco.2018.79.1905 [published Online First: 2018/10/30]

For peer review only

Figure legends

Figure 1. Rate differences and 95% confidence intervals in cancer incidence comparing women (A) and men (B) in rural versus urban areas in China, 2008-2012

Figure 2. Rate ratios and 95% confidence intervals in cancer incidence comparing women (A) and men (B) in rural versus urban areas in China, 2008-2012

For peer review only

Table 1. Cancer incidence rates by anatomic site in women in China, 2008-2012

Cancer type	Rural areas			Urban areas		
	Number of cases	Crude Rate (95% CI)	Age-standardized rate (95% CI)	Number of cases	Crude Rate (95% CI)	Age-standardized rate (95% CI)
Lip, oral cavity and pharynx	850	3.7 (3.4, 3.9)	2.8 (2.6, 3.0)	8,329	6.2 (6.1, 6.3)	4.3 (4.2, 4.4)
Esophagus	8,408	36.2 (35.4, 36.9)	26.3 (25.8, 26.9)	6,034	4.5 (4.4, 4.6)	2.7 (2.6, 2.8)
Stomach	8,410	36.2 (35.4, 36.9)	26.5 (25.9, 27.1)	22,267	16.6 (16.4, 16.8)	10.5 (10.4, 10.7)
Small intestine	162	0.7 (0.6, 0.8)	0.5 (0.4, 0.6)	1,818	1.4 (1.3, 1.4)	0.9 (0.8, 0.9)
Colon, rectum and anus	4,268	18.4 (17.8, 18.9)	13.5 (13.1, 13.9)	47,907	35.7 (35.4, 36.1)	22.3 (22.1, 22.5)
Liver and biliary passages	6,084	26.2 (25.5, 26.8)	19.2 (18.8, 19.7)	24,268	18.1 (17.9, 18.3)	11.2 (11.1, 11.3)
Pancreas	1,555	6.7 (6.4, 7.0)	4.8 (4.6, 5.0)	10,067	7.5 (7.4, 7.7)	4.6 (4.5, 4.6)
Larynx	81	0.3 (0.3, 0.4)	0.3 (0.2, 0.3)	508	0.4 (0.3, 0.4)	0.2 (0.2, 0.3)
Lung	7,208	31.0 (30.3, 31.7)	22.6 (22.0, 23.1)	54,148	40.4 (40.1, 40.7)	25.0 (24.8, 25.2)
Bone	448	1.9 (1.8, 2.1)	1.6 (1.4, 1.7)	1,665	1.2 (1.2, 1.3)	1.0 (0.9, 1.0)
Melanoma of skin	109	0.5 (0.4, 0.6)	0.4 (0.3, 0.4)	905	0.7 (0.6, 0.7)	0.4 (0.4, 0.5)
Skin, excluding melanoma	563	2.4 (2.2, 2.6)	1.7 (1.6, 1.9)	5,353	4.0 (3.9, 4.1)	2.4 (2.4, 2.5)
Mesothelioma	31	0.1 (0.1, 0.2)	0.1 (0.1, 0.1)	319	0.2 (0.2, 0.3)	0.2 (0.1, 0.2)
Breast	6,539	28.1 (27.4, 28.8)	21.5 (21.0, 22.0)	80,535	60.1 (59.7, 60.5)	41.3 (41, 41.6)
Uterus	5,572	24 (23.3, 24.6)	18.4 (17.9, 18.8)	34,763	25.9 (25.7, 26.2)	18.1 (17.9, 18.3)
Ovary	1,269	5.5 (5.2, 5.8)	4.2 (4.0, 4.5)	12,782	9.5 (9.4, 9.7)	6.7 (6.6, 6.8)
Other female genital organs	200	0.9 (0.7, 1.0)	0.7 (0.6, 0.7)	2,038	1.5 (1.5, 1.6)	1.0 (1.0, 1.1)
Kidney	380	1.6 (1.5, 1.8)	1.3 (1.2, 1.4)	5,642	4.2 (4.1, 4.3)	2.8 (2.8, 2.9)
Bladder	507	2.2 (2.0, 2.4)	1.6 (1.5, 1.7)	5,029	3.8 (3.6, 3.9)	2.3 (2.2, 2.3)
Other urinary organs	73	0.3 (0.2, 0.4)	0.2 (0.2, 0.3)	2,271	1.7 (1.6, 1.8)	1.0 (1.0, 1.1)
Eye	51	0.2 (0.2, 0.3)	0.2 (0.1, 0.3)	217	0.2 (0.1, 0.2)	0.2 (0.2, 0.2)

For peer review only - http://bmjopen.bmj.com/site/about/guidelines.xhtml

Brain and nervous system	1,405	6.0 (5.7, 6.4)	4.9 (4.6, 5.1)	7,457	5.6 (5.4, 5.7)	4.2 (4.1, 4.3)
Thyroid	999	4.3 (4.0, 4.6)	3.4 (3.2, 3.7)	22,869	17.1 (16.8, 17.3)	13.0 (12.8, 13.2)
Other endocrine organs	165	0.7 (0.6, 0.8)	0.6 (0.5, 0.7)	1,229	0.9 (0.9, 1.0)	0.7 (0.7, 0.8)
Hodgkin lymphoma	42	0.2 (0.1, 0.2)	0.2 (0.1, 0.2)	508	0.4 (0.3, 0.4)	0.3 (0.3, 0.4)
Non-Hodgkin lymphoma	862	3.7 (3.5, 4.0)	2.9 (2.7, 3.1)	7,672	5.7 (5.6, 5.9)	3.9 (3.8, 4.0)
Multiple myeloma	185	0.8 (0.7, 0.9)	0.6 (0.5, 0.7)	2,162	1.6 (1.5, 1.7)	1.0 (1.0, 1.1)
Lymphoid leukemia	1,189	5.1 (4.8, 5.4)	4.6 (4.3, 4.8)	6,565	4.9 (4.8, 5.0)	4.2 (4.1, 4.3)
Other and unspecified	961	4.1 (3.9, 4.4)	3.2 (3.0, 3.4)	13,590	10.1 (10.0, 10.3)	6.7 (6.6, 6.8)
All sites but skin	58,013	249.5 (247.5, 251.5)	186.9 (185.4, 188.4)	383,564	286.2 (285.3, 287.1)	190.9 (190.3, 191.5)
All sites	58,576	251.9 (249.9, 253.9)	188.6 (187.1, 190.2)	388,917	290.2 (289.3, 291.1)	193.4 (192.7, 194)

CI, confidence interval.

For peer review only

Table 2. Cancer incidence rate by anatomic site in men in China, 2008-2012

Cancer type	Rural areas			Urban areas		
	Number of cases	Crude Rate (95% CI)	Age-standardized rate (95% CI)	Number of cases	Crude Rate (95% CI)	Age-standardized rate (95% CI)
Lip, oral cavity and pharynx	1,517	6.3 (6.0, 6.6)	5.0 (4.8, 5.3)	18,962	14.2 (14.0, 14.4)	10.1 (10.0, 10.2)
Esophagus	13,139	54.5 (53.6, 55.4)	43.5 (42.7, 44.2)	21,961	16.5 (16.2, 16.7)	11.1 (11.0, 11.3)
Stomach	18,515	76.8 (75.7, 77.9)	61.3 (60.4, 62.2)	45,179	33.9 (33.5, 34.2)	23.0 (22.8, 23.2)
Small intestine	190	0.8 (0.7, 0.9)	0.6 (0.5, 0.7)	2,238	1.7 (1.6, 1.7)	1.2 (1.1, 1.2)
Colon, rectum and anus	5,121	21.2 (20.7, 21.8)	17.1 (16.6, 17.6)	60,205	45.1 (44.8, 45.5)	30.7 (30.4, 30.9)
Liver and biliary passages	13,885	57.6 (56.7, 58.6)	45.8 (45.1, 46.6)	56,100	42.0 (41.7, 42.4)	29.0 (28.7, 29.2)
Pancreas	1,802	7.5 (7.1, 7.8)	6.0 (5.8, 6.3)	12,593	9.4 (9.3, 9.6)	6.4 (6.3, 6.5)
Larynx	522	2.2 (2.0, 2.4)	1.7 (1.6, 1.9)	5,679	4.3 (4.1, 4.4)	2.9 (2.8, 3.0)
Lung	15,740	65.3 (64.3, 66.3)	52.7 (51.9, 53.5)	104,382	78.2 (77.7, 78.7)	52.8 (52.5, 53.1)
Bone	612	2.5 (2.3, 2.7)	2.2 (2.0, 2.4)	2,041	1.5 (1.5, 1.6)	1.2 (1.2, 1.3)
Melanoma of skin	119	0.5 (0.4, 0.6)	0.4 (0.3, 0.5)	922	0.7 (0.6, 0.7)	0.5 (0.5, 0.5)
Skin, excluding melanoma	542	2.2 (2.1, 2.4)	1.9 (1.7, 2.0)	5,611	4.2 (4.1, 4.3)	2.9 (2.8, 3.0)
Mesothelioma	30	0.1 (0.1, 0.2)	0.1 (0.1, 0.1)	477	0.4 (0.3, 0.4)	0.2 (0.2, 0.3)
Breast	68	0.3 (0.2, 0.4)	0.2 (0.2, 0.3)	677	0.5 (0.5, 0.5)	0.4 (0.3, 0.4)
Prostate	1,152	4.8 (4.5, 5.1)	4.0 (3.8, 4.3)	25,963	19.5 (19.2, 19.7)	13.0 (12.8, 13.2)
Testis	103	0.4 (0.3, 0.5)	0.4 (0.3, 0.5)	1,176	0.9 (0.8, 0.9)	0.8 (0.8, 0.9)
Other male genital organs	184	0.8 (0.7, 0.9)	0.6 (0.5, 0.7)	1,371	1.0 (1.0, 1.1)	0.7 (0.7, 0.8)
Kidney	535	2.2 (2.0, 2.4)	1.8 (1.7, 2.0)	11,201	8.4 (8.2, 8.6)	5.9 (5.8, 6.0)
Bladder	1,610	6.7 (6.4, 7.0)	5.5 (5.2, 5.7)	16,013	12.0 (11.8, 12.2)	8.1 (8.0, 8.2)
Other urinary organs	121	0.5 (0.4, 0.6)	0.4 (0.3, 0.5)	2,449	1.8 (1.8, 1.9)	1.2 (1.2, 1.3)
Eye	49	0.2 (0.2, 0.3)	0.2 (0.1, 0.3)	236	0.2 (0.2, 0.2)	0.2 (0.2, 0.2)
Brain and nervous system	1,496	6.2 (5.9, 6.5)	5.3 (5.0, 5.5)	7,454	5.6 (5.5, 5.7)	4.4 (4.3, 4.5)

For peer review only

Thyroid	293	1.2 (1.1, 1.4)	1.0 (0.9, 1.1)	7,193	5.4 (5.3, 5.5)	4.2 (4.1, 4.3)
Other endocrine organs	130	0.5 (0.5, 0.6)	0.5 (0.4, 0.5)	1,221	0.9 (0.9, 1.0)	0.8 (0.7, 0.8)
Hodgkin lymphoma	63	0.3 (0.2, 0.3)	0.2 (0.2, 0.3)	740	0.6 (0.5, 0.6)	0.5 (0.4, 0.5)
Non-Hodgkin lymphoma	1,266	5.3 (5.0, 5.5)	4.3 (4.1, 4.6)	10,025	7.5 (7.4, 7.7)	5.5 (5.4, 5.6)
Multiple myeloma	286	1.2 (1.1, 1.3)	0.9 (0.8, 1.1)	3,096	2.3 (2.2, 2.4)	1.6 (1.5, 1.6)
Lymphoid leukemia	1,433	5.9 (5.6, 6.3)	5.4 (5.1, 5.7)	8,751	6.6 (6.4, 6.7)	5.8 (5.6, 5.9)
Other and unspecified	1,186	4.9 (4.6, 5.2)	4.1 (3.9, 4.3)	16,018	12.0 (11.8, 12.2)	8.5 (8.4, 8.7)
All sites but skin	81,167	336.7 (334.4, 339.1)	271.5 (269.6, 273.4)	444,323	333 (332, 333.9)	230.5 (229.8, 231.2)
All sites	81,709	339.0 (336.7, 341.3)	273.4 (271.5, 275.2)	449,934	337.2 (336.2, 338.1)	233.4 (232.7, 234.1)

CI, confidence interval.

For peer review only - http://bmjopen.bmj.com/site/about/guidelines.xhtml

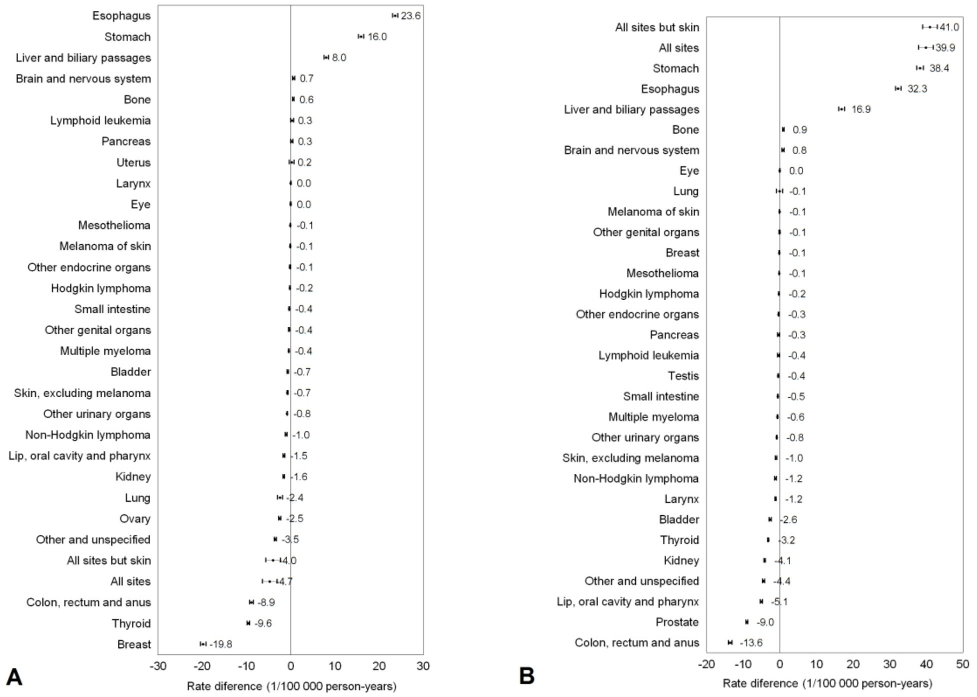

Figure 1. Rate differences and 95% confidence intervals in cancer incidence comparing women (A) and men (B) in rural versus urban areas in China, 2008-2012

721x524mm (96 x 96 DPI)

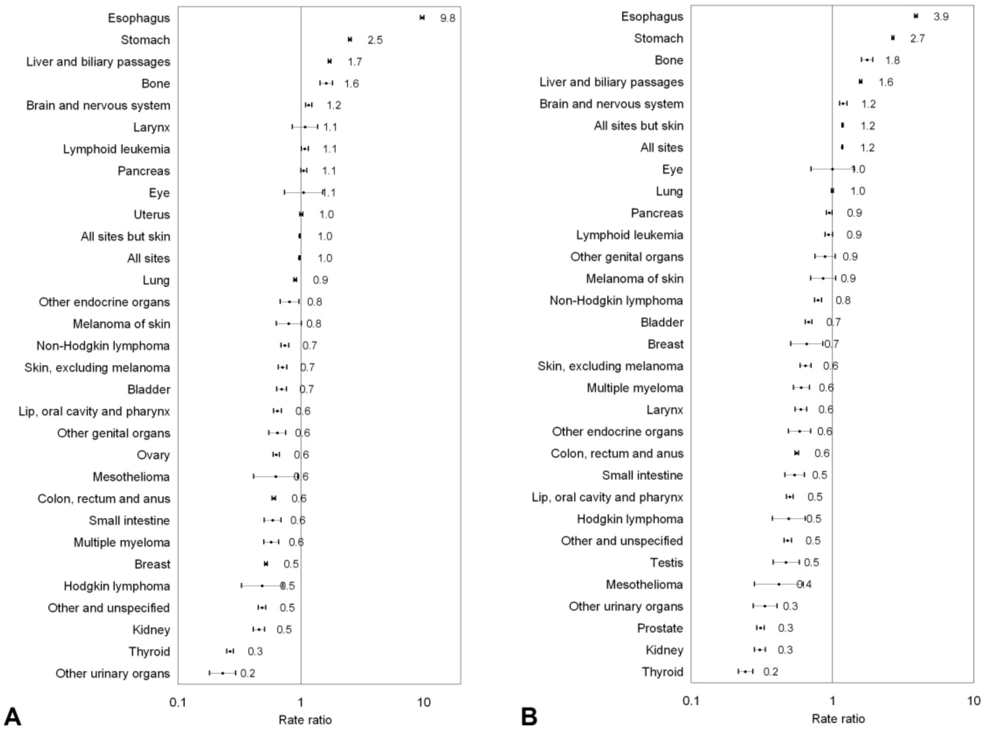

Figure 2. Rate ratios and 95% confidence intervals in cancer incidence comparing women (A) and men (B) in rural versus urban areas in China, 2008-2012

677x514mm (96 x 96 DPI)

Supporting information for

Urban-rural disparity in cancer incidence in China, 2008-2012: A cross-sectional analysis of data from 36 cancer registers

Shuai Yuan, Shao-Hua Xie

For peer review only

Supplementary Table 1. Chinese cancer registries in the Cancer Incidence in Five Continents, XI volume

Supplementary Table 2. Data quality of Chinese cancer registries in the Cancer Incidence in Five Continents, XI volume

Supplementary Table 1. Chinese cancer registries in the Cancer Incidence in Five Continents, XI volume

Region	Registry	Calendar period
Rural areas		
North	Cixian County	2008-2012
North	Shexian County	2008-2012
East	Qidong County	2008-2012
East	Jiashan County	2008-2012
East	Haimen County	2008-2012
East	Jianhu County	2010-2012
East	Guanyun	2008-2012
East	Sheyang	2008-2012
East	Xianju	2010-2012
Central	Linzhou County	2008-2012
Central	Hengdong	2009-2012
Central	Xiping	2010-2012
Central	Yanshi	2010-2012
Southwest	Yanting County	2008-2012
Urban areas		
North	Beijing	2008-2012
Northeast	Anshan City	2008-2012
Northeast	Harbin City, Nangang District	2008-2012
Northeast	Benxi	2008-2011
Northeast	Shenyang	2008-2012
East	Shanghai City	2008-2012
East	Hangzhou City	2008-2012
East	Jiaxing City	2008-2012
East	Hefei	2010-2012
East	Huaiyin District, Huai'an	2009-2012
East	Lianyungang	2008-2012
East	Maanshan	2008-2012
East	Tongling City	2008-2012
East	Wuxi	2010-2012
Central	Wuhan City	2008-2012
Central	Yueyanglou	2009-2012
South	Zhongshan City	2010-2012
South	Guangzhou	2010-2012
South	Hong Kong	2008-2012
South	Jiangmen	2010-2012
South	Liuzhou	2009-2012
South	Zhuhai	2010-2012

For peer review only

Supplementary Table 2. Data quality of Chinese cancer registries in the Cancer Incidence in Five Continents, XI volume

Region		Male				Female			
		Cases	MV (%)	DCO (%)	ASR (W)	Cases	MV (%)	DCO (%)	ASR (W)
**Rural areas**									
North	Cixian County	5,160	79.7	2.5	443.2	3,765	78.5	1.7	280.7
North	Shexian County	3,839	74.0	1.9	343.5	2,297	77.2	2.3	201.9
East	Qidong County	11,544	44.2	0.0	218.4	7,661	58.2	0.0	132.4
East	Jiashan County	4,068	64.5	0.1	224.1	2,999	73.2	0.0	169.4
East	Haimen County	10,521	48.4	0.5	209.6	7,623	62.8	0.5	154.6
East	Jianhu County	4,241	76.9	0.1	253.0	2,792	82.5	0.3	162.4
East	Guanyun	5,924	51.7	1.8	181.6	4,119	65.6	2.1	137.3
East	Sheyang	8,647	63.2	0.3	225.0	6,415	70.7	0.3	168.0
East	Xianju	2,983	64.8	4.5	277.4	1,780	69.8	4.2	174.7
Central	Linzhou County	8,721	78.4	1.5	309.8	7,111	78.2	1.3	220.5
Central	Hengdong	3,266	60.4	7.2	171.8	2,382	71.2	5.0	131.7
Central	Xiping	3,050	63.3	2.5	200.5	2,550	70.3	1.8	160.9
Central	Yanshi	2,167	67.9	3.9	215.2	1,969	69.4	4.2	170.7
Southwest	Yanting County	7,053	77.9	1.4	435.6	4,560	79.5	2.1	256.6
**Urban areas**									
North	Beijing	61,058	71.4	1.9	166.7	58,686	79.1	1.4	165.6
Northeast	Anshan City	12,562	62.4	11.8	210.5	11,581	72.6	10.5	178.8
Northeast	Harbin City, Nangang District	6,509	70.5	3.9	191.9	6,009	78.7	2.5	154.9
Northeast	Benxi	5,171	52.2	6.1	244.0	4,294	64.3	4.3	181.4
Northeast	Shenyang	26,978	61.1	6.8	181.5	25,643	69.9	5.0	157.4
East	Shanghai City	68,195	69.8	0.0	200.9	62,499	75.9	0.0	187.6
East	Hangzhou City	55,665	66.1	0.7	206.2	46,116	73.4	0.5	176.3
East	Jiaxing City	4,605	64.7	0.1	207.1	3,923	75.6	0.2	177.8
East	Hefei	10,092	52.5	8.1	252.7	6,268	58.8	6.2	155.7
East	Huaiyin District, Huai'an	5,846	62.2	8.3	220.7	3,406	70.0	7.8	130.3
East	Lianyungang	5,776	67.9	1.5	185.9	4,422	74.9	1.2	138.2
East	Maanshan	5,417	69.0	0.4	268.8	3,567	76.4	0.5	173.1
East	Tongling City	4,076	75.3	5.0	322.5	2,315	78.0	4.0	174.9
East	Wuxi	13,086	63.0	1.9	205.1	9,544	71.0	1.6	149.2
Central	Wuhan City	36,737	71.0	0.7	205.1	30,701	79.7	0.6	167.6
Central	Yueyanglou	2,396	76.1	2.9	281.0	1,795	82.5	2.8	190.8
South	Zhongshan City	6,828	70.9	0.0	251.9	4,776	83.0	0.0	158.4
South	Guangzhou	33,619	70.0	0.8	204.1	27,801	82.0	0.4	162.2
South	Hong Kong	66,837	84.0	0.7	229.8	60,084	89.7	0.6	189.9
South	Jiangmen	2,890	69.1	0.6	236.3	2,249	83.0	0.5	170.1
South	Liuzhou	5,825	62.3	2.7	224.2	4,437	75.2	2.1	165.5
South	Zhuhai	4,155	62.3	3.7	243.8	3,450	72.2	2.4	194.1

ASR, age-standardized rate; DCO, death certificate only; MV, microscopically verified.

For peer review only

STROBE 2007 (v4) Statement—Checklist of items that should be included in reports of *cross-sectional studies*

Section/Topic	Item #	Recommendation	Reported on page #
**Title and abstract**	1	(*a*) Indicate the study's design with a commonly used term in the title or the abstract	1
		(*b*) Provide in the abstract an informative and balanced summary of what was done and what was found	2
**Introduction**			
Background/rationale	2	Explain the scientific background and rationale for the investigation being reported	4
Objectives	3	State specific objectives, including any prespecified hypotheses	4
**Methods**			
Study design	4	Present key elements of study design early in the paper	5
Setting	5	Describe the setting, locations, and relevant dates, including periods of recruitment, exposure, follow-up, and data collection	5
Participants	6	(*a*) Give the eligibility criteria, and the sources and methods of selection of participants	5
Variables	7	Clearly define all outcomes, exposures, predictors, potential confounders, and effect modifiers. Give diagnostic criteria, if applicable	5
Data sources/ measurement	8*	For each variable of interest, give sources of data and details of methods of assessment (measurement). Describe comparability of assessment methods if there is more than one group	5
Bias	9	Describe any efforts to address potential sources of bias	*5*
Study size	10	Explain how the study size was arrived at	5
Quantitative variables	11	Explain how quantitative variables were handled in the analyses. If applicable, describe which groupings were chosen and why	5
Statistical methods	12	(*a*) Describe all statistical methods, including those used to control for confounding	5
		(*b*) Describe any methods used to examine subgroups and interactions	n/a
		(*c*) Explain how missing data were addressed	n/a
		(*d*) If applicable, describe analytical methods taking account of sampling strategy	n/a
		(*e*) Describe any sensitivity analyses	n/a
**Results**			

For peer review only

Participants	13*	(a) Report numbers of individuals at each stage of study—eg numbers potentially eligible, examined for eligibility, confirmed eligible, included in the study, completing follow-up, and analysed	6
		(b) Give reasons for non-participation at each stage	n/a
		(c) Consider use of a flow diagram	n/a
Descriptive data	14*	(a) Give characteristics of study participants (eg demographic, clinical, social) and information on exposures and potential confounders	6-8
		(b) Indicate number of participants with missing data for each variable of interest	n/a
Outcome data	15*	Report numbers of outcome events or summary measures	6-8
Main results	16	(*a*) Give unadjusted estimates and, if applicable, confounder-adjusted estimates and their precision (eg, 95% confidence interval). Make clear which confounders were adjusted for and why they were included	6-8
		(*b*) Report category boundaries when continuous variables were categorized	
		(*c*) If relevant, consider translating estimates of relative risk into absolute risk for a meaningful time period	n/a
Other analyses	17	Report other analyses done—eg analyses of subgroups and interactions, and sensitivity analyses	n/a
**Discussion**			
Key results	18	Summarise key results with reference to study objectives	8
Limitations	19	Discuss limitations of the study, taking into account sources of potential bias or imprecision. Discuss both direction and magnitude of any potential bias	8-9
Interpretation	20	Give a cautious overall interpretation of results considering objectives, limitations, multiplicity of analyses, results from similar studies, and other relevant evidence	8-9
Generalisability	21	Discuss the generalisability (external validity) of the study results	8-9
**Other information**			
Funding	22	Give the source of funding and the role of the funders for the present study and, if applicable, for the original study on which the present article is based	12

*Give information separately for cases and controls in case-control studies and, if applicable, for exposed and unexposed groups in cohort and cross-sectional studies.

Note: An Explanation and Elaboration article discusses each checklist item and gives methodological background and published examples of transparent reporting. The STROBE checklist is best used in conjunction with this article (freely available on the Web sites of PLoS Medicine at http://www.plosmedicine.org/, Annals of Internal Medicine at http://www.annals.org/, and Epidemiology at http://www.epidem.com/). Information on the STROBE Initiative is available at www.strobe-statement.org.

For peer review only
